# Dehydroepiandrosterone Sulfate Stimulates Expression of Blood-Testis-Barrier Proteins Claudin-3 and -5 and Tight Junction Formation via a Gnα11-Coupled Receptor in Sertoli Cells

**DOI:** 10.1371/journal.pone.0150143

**Published:** 2016-03-03

**Authors:** Dimitrios Papadopoulos, Raimund Dietze, Mazen Shihan, Ulrike Kirch, Georgios Scheiner-Bobis

**Affiliations:** Institut für Veterinär-Physiologie und -Biochemie, Fachbereich Veterinärmedizin, Justus-Liebig-Universität Giessen, Giessen, Germany; Emory University School of Medicine, UNITED STATES

## Abstract

Dehydroepiandrosterone sulfate (DHEAS) is a circulating sulfated steroid considered to be a pro-androgen in mammalian physiology. Here we show that at a physiological concentration (1 μM), DHEAS induces the phosphorylation of the kinase Erk1/2 and of the transcription factors CREB and ATF-1 in the murine Sertoli cell line TM4. This signaling cascade stimulates the expression of the tight junction (TJ) proteins claudin-3 and claudin-5. As a consequence of the increased expression, tight junction connections between neighboring Sertoli cells are augmented, as demonstrated by measurements of transepithelial resistance. Phosphorylation of Erk1/2, CREB, or ATF-1 is not affected by the presence of the steroid sulfatase inhibitor STX64. Erk1/2 phosphorylation was not observed when dehydroepiandrosterone (DHEA) was used instead of DHEAS. Abrogation of androgen receptor (AR) expression by siRNA did not affect DHEAS-stimulated Erk1/2 phosphorylation, nor did it change DHEAS-induced stimulation of claudin-3 and claudin-5 expression. All of the above indicate that desulfation and conversion of DHEAS into a different steroid hormone is not required to trigger the DHEAS-induced signaling cascade. All activating effects of DHEAS, however, are abolished when the expression of the G-protein Gnα11 is suppressed by siRNA, including claudin-3 and -5 expression and TJ formation between neighboring Sertoli cells as indicated by reduced transepithelial resistance. Taken together, these results are consistent with the effects of DHEAS being mediated through a membrane-bound G-protein-coupled receptor interacting with Gnα11 in a signaling pathway that resembles the non-classical signaling pathways of steroid hormones. Considering the fact that DHEAS is produced in reproductive organs, these findings also suggest that DHEAS, by acting as an autonomous steroid hormone and influencing the formation and dynamics of the TJ at the blood-testis barrier, might play a crucial role for the regulation and maintenance of male fertility.

## Introduction

Dehydroepiandrosterone sulfate (DHEAS) is the most abundant circulating steroid in humans. Its concentration in plasma is between 1.3 and 6.8 μM, which is about 200-fold higher than the plasma concentrations of dehydroepiandrosterone (DHEA) (7–31 nM) [1]. DHEAS is produced mainly in the adrenal zona reticularis. It is derived from DHEA, which is almost entirely converted to DHEAS by a sulfotransferase. The sulfated steroid is then secreted into the serum [2].

Sulfated steroids like DHEAS have long been considered to be physiologically inactive waste products of steroid hormone metabolism. Nevertheless, the identification of cytosolic steroid sulfatases able to hydrolyze the sulfate from the steran moiety prompted the new idea that sulfated steroids constitute a reservoir that upon desulfation can serve as precursors for the biosynthesis of other biologically active steroid hormones. In analogy, DHEAS has been considered to be a pro-androgen that has to be converted into testosterone or other steroid hormones in order to exert its biological activity [3].

This assumption, however, is not consistent with the results of various newer investigations demonstrating specific actions of DHEAS that are distinct from the actions of DHEA. Thus, 1 μM DHEAS was shown to inhibit proliferation of pheochromocytoma PC12 cells induced by nerve growth factor and to stimulate chromogranin A expression and catecholamine release from nerve growth factor -treated cells [4, 5]. In another study, DHEAS was shown to specifically stimulate growth factor-induced proliferation of bovine chromaffin cells [6], whereas DHEA had opposite effects on the growth factor responses, indicating that the cellular effects of DHEA and DHEAS are mediated through different signaling pathways [6]. The neuroprotective effects of DHEA and DHEAS might also be the result of different signaling pathways specifically triggered by either of the steroids [7]. Thus, DHEA prevents N-methyl-D-aspartate (NMDA)-induced neurotoxicity by inhibiting the NMDA receptor-induced activation of Ca^2+^-sensitive nitric oxide synthase and nitric oxide production, while the neuroprotective effects of DHEAS against NMDA-induced neurotoxicity are mediated through the Sig-1R receptor [8].

Although DHEAS is produced not only in the adrenal cortex and brain [9] but also in the gonads [10–12], surprisingly little is known about the effects of the steroid on the various cells of the reproductive system. Therefore, by using the Sertoli cell line TM4 as a model, we address in the present investigation the possibility that DHEAS has hormone-like effects by searching for signaling cascades that might be induced by the sulfated steroid. We further address a possible physiological significance of such signaling events by examining their effects on the expression of proteins that are important for male reproduction. This group of proteins includes the tight junction (TJ)-forming proteins claudin-3 and -5, whose expression is regulated by the CRE sequence and which, as components of the blood-testes barrier (BTB) [13–16], are critical for the maturation of spermatocytes. Finally, we make a first attempt to classify the DHEAS receptor within a group of known hormone receptors.

## Materials and Methods

### Cell culture

The Sertoli cell line TM4 [17] was cultured in DMEM/F-12 high glucose with 2 mM L-glutamine (Thermo Fischer Scientific, Waltham, MA, USA) supplemented with 10% standardized fetal bovine serum (FBS) (Biochrom GmbH, Berlin, Germany) and 1% penicillin/streptomycin combination (5000 units penicillin and 5 mg streptomycin/ml) (Sigma-Aldrich, St. Louis, MO, USA). Cells were incubated in a humidified incubator at 37°C under 5% CO_2_. The medium was renewed every three days.

### Detection of Sertoli cell-specific markers in TM4 cells by RT-PCR

The Sertoli cell nature of the TM4 cells was confirmed by the detection of the Sertoli cell-specific markers Sox9, ABP (androgen binding protein), and Dhh (Desert Hedgehog) via RT-PCR. TM4 cells were grown as stated above and then total mRNA was isolated by following the protocol of the commercially available SV Total RNA Isolation System (Promega, Mannheim, Germany; cat. # Z3100). The reverse transcription of the isolated mRNA was carried out by the Reverse Transcription System (Promega; cat. # A3500) according to the protocol of the provider.

For PCR amplification a total of 1.5 μg of cDNA from the reverse transcription was incubated with 20 pmol/ml of each primer (see below), 1X of Buffer BD, 2.5 mM MgCl_2_, 0.2 mM dNTPs and 1.25 units *Taq* DNA polymerase (Bio&SELL e.K., Nürnberg, Germany). The final volume of the solutions was 25 μl. PCR was carried out in a MasterCycler Gradient (Eppendorf, Hamburg, Germany). Samples were incubated at 95°C for 5 min, followed by 36 cycles of denaturation at 95°C for 1 min, annealing at 60°C for 30 sec, and cDNA extension at 72°C for 1 min. After amplification, a final extension at 72°C was performed for 10 min. The same protocol was used for all amplificates mentioned bellow.

Sox9 was identified by using as primers the oligonucleotides 5’CCAGCAAGAACAAGCCACAC3’ and 5’GCTCAGTTCACCGATGTCCA3’. They amplify a fragment of 545 bp localized between bases 668 and 1212 of mouse Sox9-specific mRNA.

ABP was identified by using the primers 5’TGGGTGGATGGGAAGGAGAT3’ and 5’CCACGCTTTGGTTTTGGAGG3’ to amplify a fragment of 430 bp between bases 592 and 1021 of mouse ABP-specific mRNA.

For the detection of Dhh the primers used were 5’AGTAGGTTCCAGGTTCCCCA3’ and 5’GGTTGCGGGACTCGTAGTAG3’. They amplify a 730 bp fragment between bases 80 and 809 of mouse Dhh-specific mRNA.

### Preparation of cell lysates

TM4 cells were seeded at a density of 2·10^5^ cells in 5-cm culture dishes and grown as described above until they reached ~70% confluence. Cells were then incubated for 24 h with 0.5% FBS. Various concentrations of DHEAS dissolved in ethanol were added to the cells and incubation was continued for 120 min. The concentration of ethanol (0.1% v/v) was identical in all samples. The medium was then removed by aspiration and cells were washed once with ice-cold phosphate-buffered saline (PBS; without Ca^2+^ or Mg^2+^; GE Healthcare, Munich, Germany) and lysed in 400 μl of a commercially available cell lysis buffer (New England Biolabs GmbH, Frankfurt, Germany; cat. # 9803) according to the protocol of the provider. The lysis buffer contains the phosphatase inhibitors vanadate and leupeptin. Immediately before use, 1 μM PMSF (Carl Roth GmbH, Karlsruhe, Germany) was added to the lysis buffer. All lysis steps were carried out on ice. After 5 min of incubation cells were harvested with a scraper, transferred into plastic tubes, and sonicated 5 times for 5 sec each. The reaction tubes were then centrifuged at 13,000 x g for 15 min at 4°C. The protein content of the supernatants was determined at 540 nm using the bicinchoninic acid (BCA) protein assay reagent kit (Pierce, Rockford, IL, USA) and a Labsystems (Helsinki, Finland) plate reader. The lysis buffer was included in the bovine serum albumin protein standard. Aliquots of the supernatant were taken and stored at −20°C for further analysis.

### Protein electrophoresis and western blotting

A total of 8 μg cell lysate protein for p-Erk1/2, total Erk1/2 and actin detection and 12 μg cell lysates protein for p-CREB and p-ATF-1 detection were separated by sodium dodecylsulfate-polyacrylamide gel electrophoresis on slab gels containing a 30% acrylamide and bis-acrylamide solution in a 37.5:1 ratio. Strep-tagged molecular weight markers (Bio-Rad Laboratories GmbH, Munich, Germany) were used to determine the relative molecular mass of the separated proteins. After electrophoresis proteins were blotted onto hydrophobic PVDF transfer membranes (Merck Chemicals GmbH, Schwalbach, Germany) for 30 min at 0.5 V/cm^2^. The membranes were then blocked for 1 h by incubation in TBS-T containing 5% nonfat milk. Desired protein bands were visualized by incubating the membranes with the primary antibody according to the protocol of the providers (Table 1) and subsequently the appropriate secondary antibody of an enhanced chemiluminescence solution (made by mixing the buffer with p-coumaric acid, luminol, and H_2_O_2_ [18]). For the simultaneous detection of phospho-CREB and phospho-ATF-1, western blots were probed with an antibody that cross-reacts with the two phosphorylated proteins (Cell Signaling Technology). A Strep-Tactin and horseradish peroxidase-conjugate (Bio-Rad Laboratories) at a dilution of 1:20,000 was included in the mixture containing the secondary antibody in order to detect the Strep-tagged molecular weight marker. The chemiluminescence obtained was visualized by exposure to film. Films were analyzed by the TotalLab gel image analysis software (Biostep, Jahnsdorf, Germany).

**Table 1:** Antibodies used in the study and their providers.

Antibody	Catalog no.	Application	Provider	Address
Anti-total Erk1/2	9102	Western Blot	Cell Signaling Technology	Frankfurt am Main, Germany
Anti-pan-Actin	4968	Western Blot	Cell Signaling Technology	Frankfurt am Main, Germany
Anti-phospho-Erk1/2	4370	Western Blot, Immunofluorescence	Cell Signaling Technology	Frankfurt am Main, Germany
Anti-phospho-CREB	9198	Western Blot, Immunofluorescence	Cell Signaling Technology	Frankfurt am Main, Germany
Anti-phospho-ATF-1	Ab76085	Immunofluorescence	Abcam	Cambridge, United Kingdom
Anti-claudin 3	NBP1-67517	Immunofluorescence	Novus Biologicals	Cambridge, United Kingdom
Anti-claudin 5	Sc-28670	Immunofluorescence	Santa Cruz Biotechnology, Inc.	Heidelberg, Germany
Anti-Gnα11	Sc-390382	Immunofluorescence	Santa Cruz Biotechnology, Inc.	Heidelberg, Germany
Anti-AR	Sc-13062	Immunofluorescence	Santa Cruz Biotechnology, Inc	Heidelberg, Germany

The PVDF membranes were occasionally reused for the detection of additional proteins. Primary and secondary antibodies were thus stripped by incubating the membrane 3 times for 15 min in 50 ml of 100 mM glycine, pH 2.0. To ensure the successful removal of the antibodies, the membranes were incubated in the enhanced chemiluminescence solution and controlled for any residual fluorescence signals by exposure to film for 5 min. The membranes were then washed once in 25 ml TBS-T for 5 min. After blocking in 5% nonfat milk, western blotting for the detection of further proteins of interest were carried out as described above.

### Detection of specific mRNA/cDNA for claudin-3, claudin-5, Gnα11, Gnαq, and glyceraldehyde-3-phosphate dehydrogenase (GAPDH) by PCR

For total mRNA isolation, TM4 cells were seeded at a density of 3·10^5^ cells in 5-cm culture dishes and grown to a confluence of 80–90%. Afterwards, the medium was replaced with one including 2% FBS, 1% penicillin/streptomycin, and 1 μM DHEAS. The cells were incubated in this medium for 48 h and total mRNA was then isolated and reversed-transcribed into cDNA as described above. PCR amplification was carried out as described above, with the following differences:

Claudin-3 specific mRNA/cDNA was amplified in 38 PCR cycles. The annealing temperature was 60°C. The forward primer for claudin-3 was the oligonucleotide 5´GTACAAGACGAGACGGCCAA3´ and the reverse primer was 5´TTCCAGCCTAGCAAGCAGAC3´. These amplify a region between bases 550 and 1033 of mouse claudin 3-specific mRNA and yield an amplificate of 484 bp.

For PCR amplification of claudin-5, a similar protocol was used with the exception that a total of 2 μg of cDNA was used. The annealing temperature was 57°C. The forward primer for claudin-5 was the oligonucleotide 5´GAGTTCAGCTTCCCGGTCAA3´ and the reverse primer was 5´TGCCCTTTCAGGTTAGCAGG3´. These amplify a region between bases 726 and 1176 of mouse claudin-5-specific mRNA and yield an amplificate of 451 bp.

GAPDH-specific mRNA/cDNA by using as forward primer the oligonucleotide 5´GGAGATTGTTGCCATCAACG3´ and as reverse primer the oligonucleotide 5´CACAATGCCAAAGTTGTCA3´. These amplify a fragment of 430 bp between bases 128 and 557 of mouse GAPDH-specific mRNA.

Gnα11-specific mRNA/cDNA was amplified under the same conditions as GAPDH but with an annealing temperature of 54°C. Forward and reverse primers were the oligonucleotides 5´GAACCGGGAAGAGGTAGGG3´ and 5´GACCAAGTGTGAGTGCAGGA3´, respectively. These amplify a 917-bp fragment of mouse Gnα11-specific mRNA localized between bases 70 and 986.

Gnαq-specific mRNA was amplified under the same conditions as the Gnα11-specific mRNA. Forward and reverse primers were the oligonucleotides 5´TGAGTGAGCCTGTCAAGCAG´ and 5´AAGCTCTGACGGATGGTGTG´, respectively. These amplify a 688-bp fragment of mouse Gnαq-specific mRNA localized between bases 3239 and 3926.

### Silencing expression of Gnα11 or nuclear AR via siRNA

Silencing of the Gnα11 protein was carried out by using commercially available siRNA and by following the protocol of the provider (Silencer® Select siRNA; Invitrogen, Karlsruhe, Germany). The oligonucleotides used were 5´CAAGAUCCUCUACAAGUAUTT3´ and 5´AUACUUGUAGAGGAUCUUGAG3´ at a concentration of 50 nM each. Control cells were treated with OptiMem plus Lipofectamine RNAiMAX alone or OptiMem plus Lipofectamine RNAiMAX plus the siRNA Negative Control as supplied by the provider. Transfection efficiencies in the range of 88±6% were estimated by the Block-iT^TM^ Transfection Kit (Invitrogen) according the protocol of the provider.

The expression of AR was silenced by using commercially available siRNA and by following the protocol of the provider (Stealth^TM^ RNAi; Invitrogen). The oligonucleotides used were 5´ACUCGAUCGCAUCAUUGCAUGCAAA3´ and 5´UUUGCAUGCAAUGAUGCGAUCGAGU3´. Control cells were treated with Stealth^TM^ RNAi Negative Control, as provided in the kit. Transfection efficiency was estimated by the Block-iT^TM^ Transfection Kit.

TM4 cells were seeded at a density of 3·10^4^ cells in 6-well plates (Greiner, Frickenhausen, Germany) and siRNA treatment and cell propagation were carried out following the protocol of the provider. After treatment, preparation of samples for PCR, western blots, or immunofluorescence experiments was carried out as described in the previous or subsequent paragraphs.

### Immunofluorescence

For the detection of phosphorylated proteins TM4 cells at 60–70% confluence were incubated with 1 μM DHEAS for 120 min. For the detection of claudins cells at 80% confluence were incubated with 1 μM DHEAS for 48 hours. Detection of Gnα11 was carried out similarly to the detection of claudins (see bellow). All immunofluorescence experiments were carried out in 2-well chamber slides (Sigma-Aldrich). Unless otherwise specified the following procedures were carried out at room temperature. All incubation steps with UV-sensitive reagents were performed in the dark.

For the detection of active Erk1/2 following DHEAS incubation, the medium was aspirated and the cells were fixed using 450 μl of ice-cold methanol containing a total of 20 ng of DAPI (4',6-diamidino-2-phenylindole). After 15 min of incubation at RT, the DAPI solution was aspirated and slides were allowed to dry for 15 min. The cells were then blocked with 10% FBS and 0.3% Triton-X100 in PBS for 1 h. The slides were then washed twice with 500 μl PBS. Afterwards, PBS containing 2% FBS and 0.3% Triton with the primary antibody against phosphorylated Erk1/2 (Table 1) at a 1:200 ratio was added to the slides, and incubation was continued for 2 days at 4°C in a humidified chamber. Staining was achieved by incubating for 45 min at room temperature with an Alexa Fluor 488-labelled goat anti-rabbit IgG (Invitrogen, Karlsruhe, Germany) diluted at 1:500 in 2% FBS, 0.3% Triton-X100 in PBS. The solution was then removed, the cells were washed twice with PBS and afterwards 1 ml PBS was added and samples were stored at 4°C for further investigation.

For the detection of active CREB and ATF-1 following incubations, the DHEAS-containing medium was aspirated and cells were fixed in 3.7% formaldehyde for 15 min. The formaldehyde solution was then removed, the slides were washed twice with 500 μl PBS, and cells were incubated in blocking solution (3% BSA/0.6% Triton X-100 in Tris-buffered saline, TBS) for 1 h. Thereafter, the incubation medium was replaced by TBS containing 1% BSA, 0.6% Triton X-100, and the primary antibodies against phosphorylated ATF-1 and phosphorylated CREB (Table 1) at a dilution of 1:150 and 1:200, respectively. Incubation continued for 48 h at 4°C in a humidified chamber. For fluorescence staining, the incubation medium was aspirated, the slides were washed twice with PBS, and then TBS containing 1% BSA, 0.6% Triton X-100, and the secondary antibody (goat anti-rabbit IgG) labeled with AlexaFluor 488 at a dilution of 1:250 was added. Incubation continued for 1 h at room temperature. The solution was then removed and the cells were washed twice with PBS. To stain the nuclei, cells were incubated for 10 min in methanol containing 0.5 μg/ml DAPI. The methanol was aspirated, 1 ml PBS was added, and samples were stored at 4°C for further investigation.

The determination of total protein expression of claudin-3 and claudin-5, Gnα11, and AR was carried out using the same protocol as that used for the transcription factors CREB and ATF-1 with a few minor modifications. The blocking buffer used was TBS containing 3% BSA and 0.3% Triton X-100 and the antibody dilution buffer used was TBS containing 1% BSA and 0.3% Triton X-100. The primary antibodies (Table 1) were added to the antibody dilution buffer at a dilution of 1:200. The secondary antibody, labeled with AlexaFluor 488, was added at a dilution of 1:300 to the antibody dilution buffer.

All images were obtained using an inverse Olympus IX81 microscope equipped with the corresponding fluorescence system (Olympus, Hamburg, Germany). Fluorescence of the Alexa Fluor 488-labelled secondary antibody within cells was measured by using the software program ImageJ (freely available at http://rsbweb.nih.gov/ij/). Only green fluorescence indicating phosphorylated Erk1/2, CREB, or ATF-1 or total Gnα11, claudin-3 or claudin-5 was considered. A total of 90 cells closest to the diagonals of the optical field from three similar experiments were considered. Measurements of fluorescence were carried out by following the protocol of ImageJ (http://www.slu.se/PageFiles/388774/Pacho%20ImageJ%20measuring-cell-fluorescence.pdf). Data points were transferred to and analyzed by the software program GraphPad Prism4 (GraphPad Software Inc., La Jolla, CA, USA).

### Inhibition of steroid sulfatase by STX64

Cells were incubated as described above with or without 100 nM DHEAS in the presence or absence of 10 nM STX64 (Sigma-Aldrich, Taufkirchen, Germany), a concentration that is considered sufficient for complete inactivation of steroid sulfatases [19]. All samples contained 0.1% DMSO (v/v), which was the solvent for stock preparations of STX64. After 120 min of incubation cells were subjected to a fixation/immunostaining procedure as described in the previous paragraph in order to detect activated Erk1/2.

### Measurement of transepithelial resistance (TER)

A total of 8 x 10^4^ cells/cm^2^ were seeded on ThinCert inserts with a 0.4-μm pore diameter for use with 24-well plates (both from Greiner) and cultured as described above. After confluence was reached incubation in fresh media was carried out for the indicated periods of time in the presence or absence of 1 μM DHEAS. Measurement of TER was carried out with a Millicell ERS-2 epithelial Volt-Ohm meter (Merck Millipore, Darmstadt, Germany). TER in Ω x cm^2^ was calculated according to the protocol of the Volt-Ohm meter manufacturer and by considering the resistance of cell-free filters. The electrode of the Volt-Ohm meter was mounted on an adjustable stand to ensure its precise depth positioning in each of the wells.

### Statistical analysis

Loading differences in the various western blots were corrected by taking into consideration the optical density of unphosphorylated Erk1/2 bands or total actin bands, detected in western blots that were run in parallel. Data were analyzed by GraphPad Prism4 software and by applying one-way ANOVA with repeated measures and Dunnett's comparison of all data with the control. Differences were considered to be significant when p<0.05. Immunofluorescence experiments were repeated at least three times. The values used in the statistical analysis are listed under S1 Table.

## Results

### Verification of Sertoli cell characteristics of TM4 cells

The Sertoli cell nature of the TM4 cells was confirmed by the detection of the Sertoli cell-specific markers Sox9 (regulates the differentiation of Sertoli cells in the testis [20, 21]), ABP (androgen binding protein; a functional marker of Sertoli cells [22, 23]), and Dhh (Desert Hedgehog; regulates the male germ line [24]) via RT-PCR (S1 Fig). Based on these findings, and since the TM4 cells also express the classical androgen receptor (AR; see below), we concluded that this cell line constitutes a reliable model for studying Sertoli cell function.

### DHEAS induces activation of Erk1/2, CREB, and ATF-1 in TM4 cells: evidence from immunofluorescence studies

In the spermatogenic cell line GC-2 interaction of DHEAS with a membrane-bound receptor activates the Src/Ras/Raf/Erk1/2 signaling cascade and stimulates the transcription factors CREB (cyclic AMP-responsive element binding protein) and ATF-1 (activating transcription factor-1) [25]. Both CREB and ATF-1 are members of the bZIP superfamily of transcription factors and stimulate transcription when activated by phosphorylation at either Ser63 (ATF-1) or Ser133 (CREB). Thus, our first aim in the current investigation was to examine whether DHEAS might also induce Erk1/2, CREB, and ATF-1 activation in the Sertoli cell line TM4.

**Fig 1:**
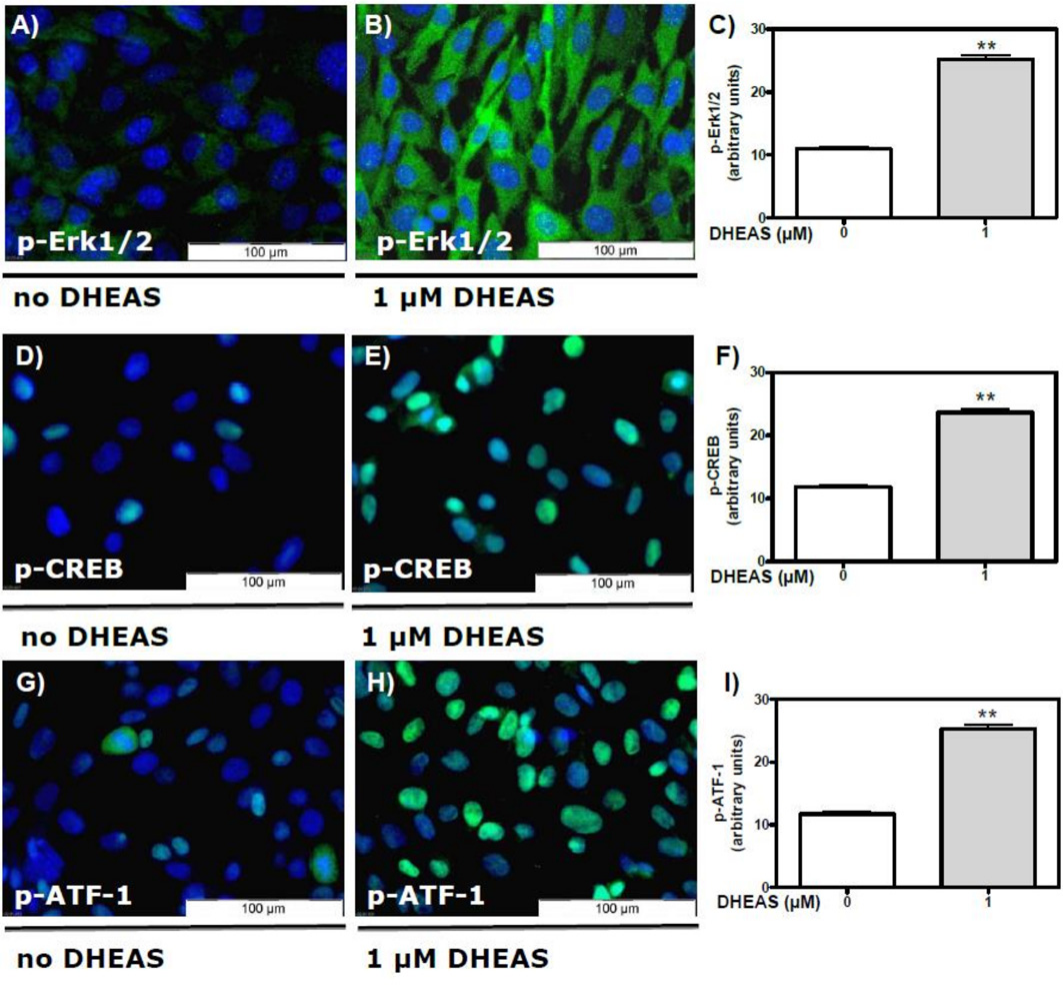
**Activation of Erk1/2 and transcription factors CREB and ATF-1 by DHEAS in TM4 cells detected by immunofluorescence:** TM4 cells were incubated with or without 1 μM DHEAS for 120 min and then stained with specific antibodies against either phospho-Erk1/2 (A-B), phospho-CREB (D-E) or phospho-ATF-1 (G-H) and a secondary Alexa Fluor 488-labeled antibody as described under “Methods”. Green fluorescence indicates the phosphorylation of Erk1/2 (A-B), CREB (D-E) of ATF-1 (G-H). Nuclei of the cells were labeled with DAPI and appear blue. (A, D and G) Fluorescence of phospho-Erk1/2, phospho-CREB or phospho-ATF-1 in the absence of DHEAS. (B, E and H) Phosphorylation of Erk1/2, CREB and ATF-1 in response to DHEAS (1 μM) treatment. (C, F and I) Statistical analysis of the corresponding green fluorescence in arbitrary units (n = 90; means ±SEM; **p≤0.01).

TM4 cells were incubated with either 0 or 1 μM DHEAS and then subjected to a fixation/immunostaining procedure as stated under “Methods”. Phosphorylated forms of Erk1/2, ATF-1, or CREB were detected by using appropriate antibodies (Table 1). Activation of Erk1/2 in the absence of DHEAS was negligible (Fig 1A). Incubation of the cells with 1 μM DHEAS resulted in the stimulation (phosphorylation) of Erk1/2 in every cell within the optical field (Fig 1B) in a highly significant fashion (Fig 1C).

Activation of Erk1/2 leads to subsequent activation of various transcription factors, among them the transcription factors CREB and ATF-1 [25–28]. In the absence of DHEAS, active CREB (Fig 1D) or ATF-1 (Fig 1G) were barely detectable. Treatment of cells with 1 μM DHEAS for 120 min led to a significant activation of CREB (Fig 1E and 1F) or ATF-1 (Fig 1H and 1I), consistent with the observed DHEAS-induced Erk1/2 activation. Whereas the latter appeared as green fluorescence spread over the entire area of the DHEAS-treated cells (Fig 1B), activated transcription factors CREB and ATF-1 were visible as expected as green fluorescent signals within the nuclei (Fig 1E and 1H).

### Detection of phospho-Erk1/2, phospho-CREB, and phospho-ATF-1 in western blots

**Fig 2:**
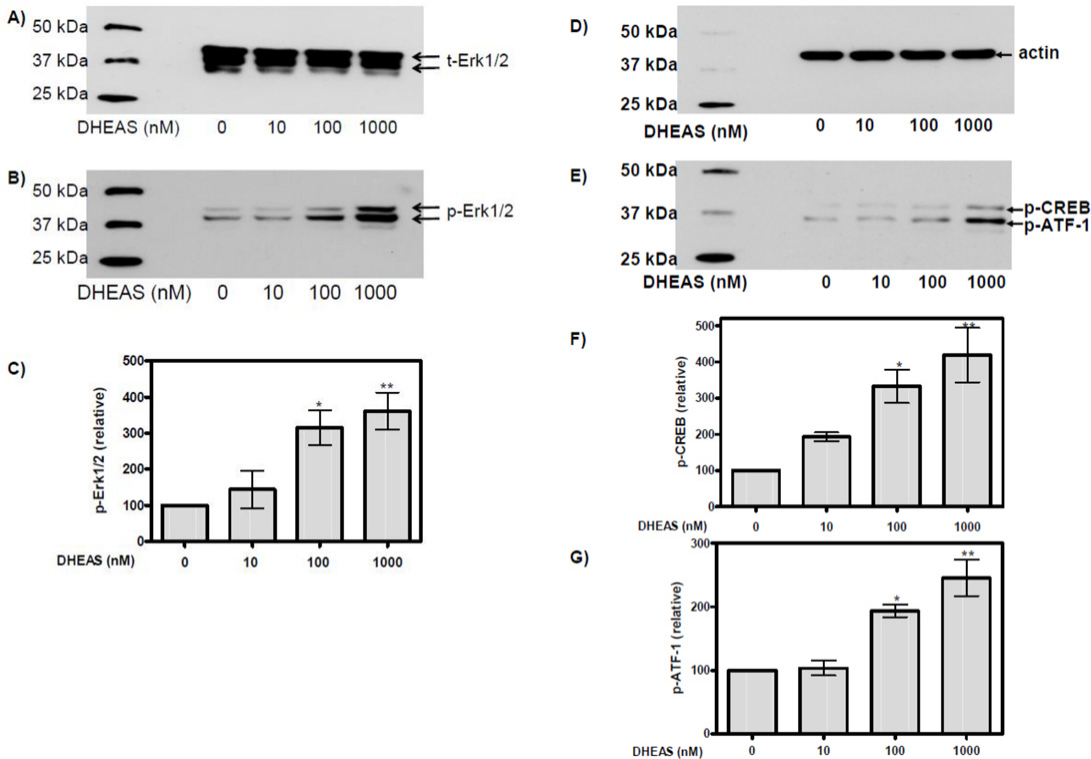
DHEAS-induced activation of Erk1/2, CREB and ATF-1 detected in western blots. TM4 cells were treated for 120 min with the indicated concentrations of DHEAS. Proteins in cell lysates were then separated on SDS polyacrylamide gels and subsequently probed in a western blot using a monoclonal antibody against either total Erk1/2 (t-Erk1/2) (A), as a loading control, or phosphorylated (activated) Erk1/2 (p-Erk1/2) (B). The western blots in (A) and (B) show typical results for the Erk1/2 bands of 42/44 kDa. (C) Statistical analysis of Erk1/2 activation as a function of DHEAS concentration in several identical experiments in which the chemiluminescence was quantified by gel image analysis software (n = 4; mean ±SEM; *p≤0.05; **p≤0.01). (D) Detection of total actin served as loading control in further western blots for the detection of either phosphorylated CREB or ATF-1 (E). The western blots in (D) and (E) show representative results from several identical experiments using the indicated concentrations of DHEAS; the quantification and statistical analysis of these results are shown in (F) and (G) (n = 4; mean±SEM; *p≤0.05; **p≤0.01).

Since immunofluorescence can only reliably detect activated Erk1/2 and transcription factors CREB and ATF-1 within cells residing in the optical field of the microscope, we carried out western blot experiments to obtain a representative average of DHEAS actions on all cells in the incubation mixture. After 120 min of incubation DHEAS induced a concentration-dependent stimulation (phosphorylation) of Erk1/2 (Fig 2B) without affecting the total amount of this kinase (Fig 2A). Activation was significant at DHEAS concentrations above 100 nM (Fig 2C); at 1 μM DHEAS, the amount of phosphor-Erk1/2 was more than three-fold higher than in the absence of the steroid.

Use of an antibody that cross-reacts with phospho-CREB and phospho-ATF-1 (Table 1) demonstrated a DHEAS concentration-dependent stimulation (phosphorylation) of both transcription factors (Fig 2E) after an incubation period of 120 min. DHEAS had no effect on actin expression (Fig 2D), indicating that the observed changes in phospho-CREB or phospho-ATF-1 were not due to a general increase in protein expression. As with Erk1/2, activation of CREB or ATF-1 was significant at DHEAS concentrations above 100 nM (Fig 2F and 2G) and 2.5 or 4.5 times higher, respectively, than the basal activity of these factors in the absence of the steroid.

### DHEAS does not need to be converted to DHEA or testosterone in order to induce phosphorylation of Erk1/2

A cellular steroid sulfatase could potentially convert DHEAS to its analogue DHEA and facilitate further modification to testosterone. In order to assess these possibilities, we investigated a) whether the sulfatase inhibitor STX64 influences DHEAS-induced phosphorylation of Erk1/2, b) whether DHEA under the conditions applied for DHEAS might also induce Erk1/2 phosphorylation, and c) whether abrogation of AR expression might prevent activation of Erk1/2 by DHEAS.

**Fig 3:**
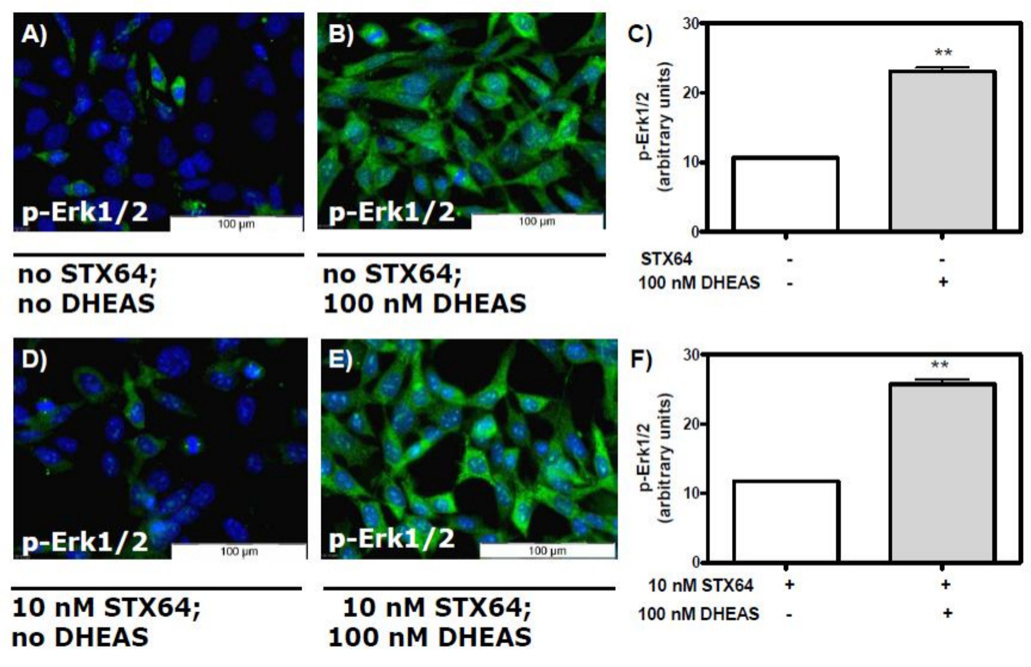
Stimulation of Erk1/2 phosphorylation by DHEAS in the presence of the steroid sulfatase inhibitor STX64. Cells were incubated with or without 100 nM DHEAS in the presence or absence of 10 nM STX64. Nuclei of the cells were labeled with DAPI and appear blue. (A and D) Fluorescence in the absence of DHEAS. (B and E) Phospho-Erk1/2 fluorescence after stimulation with 100 nM DHEAS in cells that had been pre-treated without (B) or with (E) 10 nM STX64. (C) Statistical analysis of multiple experiments identical to those shown in (A) and (B) (n = 90; means ±SEM; **p≤0.01). (F) Similar results were obtained in the presence of STX64 for results shown in panels (D) and (E) (n = 90; means ±SEM; **p≤0.01).

In order to determine whether sulfatase activity is essential for the DHEAS-induced activation of Erk1/2, we repeated the immunofluorescence experiments described above in the presence of the steroid sulfatase inhibitor STX64. The concentration of inhibitor chosen was 10 nM, which is reported to be sufficient for complete inactivation of the steroid sulfatase [19]. Similar concentrations are also used for the treatment of breast cancer [29]. The DHEAS concentration used in these experiments was 100 nM, which was 10-fold lower that the concentration used for the results shown in Figs 1 and 2. The presence of STX64 did not affect the DHEAS-induced activation of Erk1/2 (Fig 3). Thus, a conversion of DHEAS to DHEA and subsequently to other steroid hormones does not appear to be a prerequisite for Erk1/2 activation.

**Fig 4:**
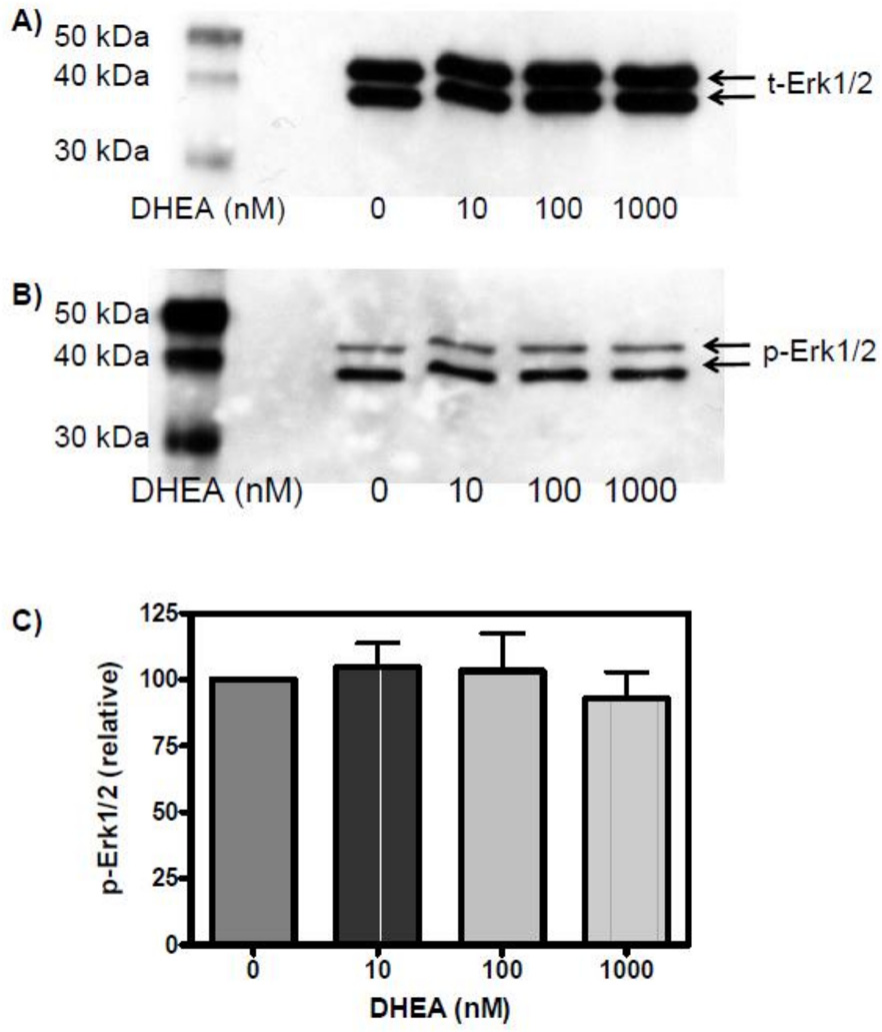
Effect of DHEA on Erk1/2 phosphorylation. The conditions are the same as those used in Fig 2, with the exception that DHEA was used instead of DHEAS. (A) The detection of total Erk1/2 served as loading control and was carried out after stripping of the antibodies used in (B) to detect phosphorylated Erk1/2. (C) Statistical analysis of Erk1/2 activation as a function of DHEA concentration (n = 3; mean ±SEM).

This conclusion was further supported through experiments addressing the direct effects of DHEA on Erk1/2 activation. Incubation of cells with DHEA under the same conditions as with DHEAS did not result in any phosphorylation of Erk1/2 above that observed in the untreated controls (Fig 4).

**Fig 5:**
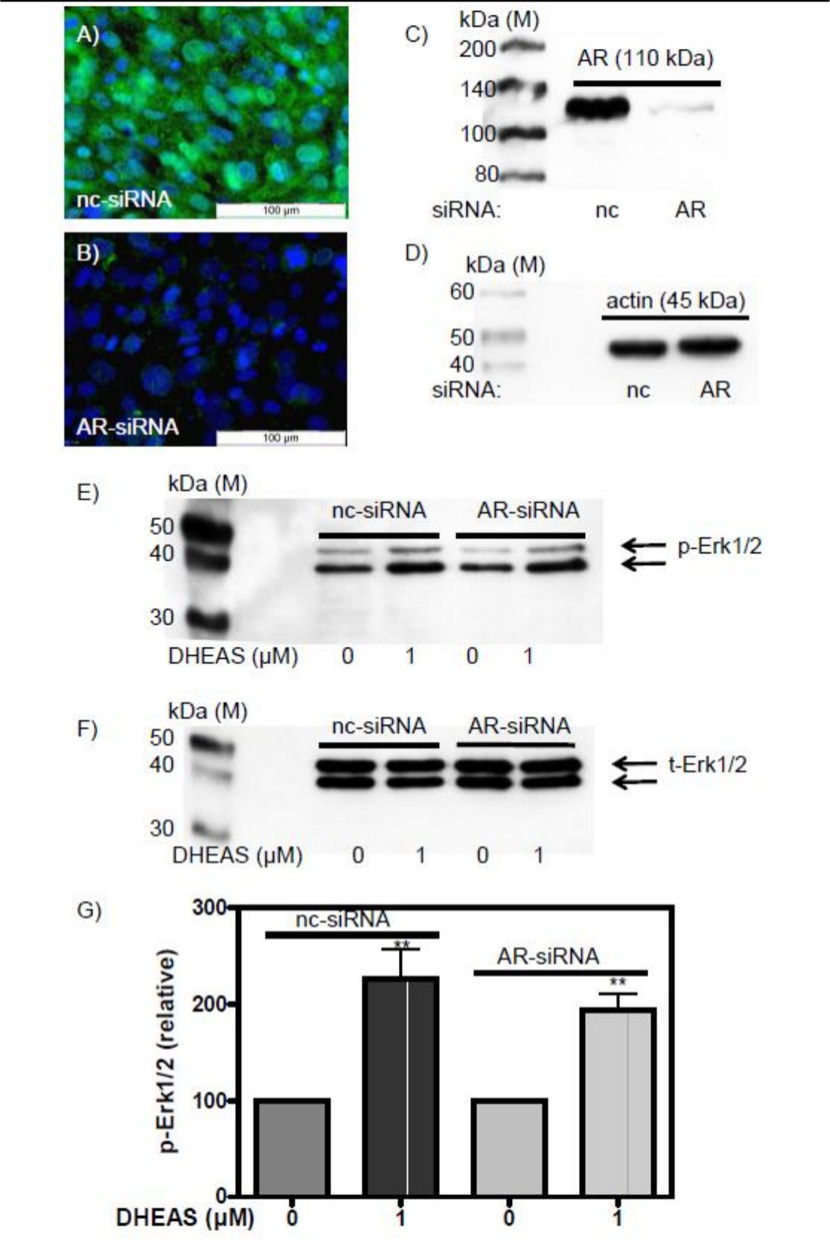
DHEAS-induced phosphorylation of Erk1/2 after silencing AR expression by siRNA. (A) AR expression, indicated by the green fluorescence of the secondary antibody, in TM4 cells exposed to negative control siRNA (nc-siRNA). (B) Abrogation of AR expression after treatment of the cells with AR-specific siRNA (AR-siRNA). In both A and B, nuclei are stained blue. (C) Western blot showing that the expression of AR in the presence of AR-siRNA is reduced by 94 ± 5% (n = 3). (D) At the same time expression of actin is not affected by AR-siRNA, indicating that the reduction of AR in (C) is specific and not due to an overall suppression of protein expression. (E) Erk1/2 phosphorylation in response to DHEAS after abrogation of AR expression with AR-specific siRNA. (F) Total Erk1/2 levels after treatment of cells with either nc-siRNA or AR-siRNA. The western blot shown was generated from the western blot shown in (E) which was first stripped of the original antibodies and then reprobed with appropriate antibodies to detect total Erk1/2. (G) Statistical analysis of Erk1/2 activation in the presence of either nc-siRNA or AR-siRNA (n = 3; mean ±SEM; **p≤0.01).

Finally, since phosphorylation of Erk1/2, CREB, and ATF-1 are also triggered by testosterone via the so-called non-classical pathway of testosterone signaling, and because DHEAS might be converted to testosterone in our incubations, we examined DHEAS-induced effects after abrogation of AR expression by means of siRNA (Fig 5A–5D). Under these conditions there was no significant difference in DHEAS-stimulated Erk1/2 activation compared with controls that had been treated with nc-siRNA (Fig 5E–5G).

All of these data indicate that DHEAS does not need to be converted into DHEA or testosterone to stimulate Erk1/2 phosphorylation. The data instead are consistent with the idea that DHEAS itself acts as a hormone.

### Stimulation of claudin-3 and claudin-5 expression by DHEAS

Various claudins are expressed in rodent testes [30, 31]. Among them claudin-3 and claudin-5 are proteins involved in the formation of TJ and therefore crucial components of the BTB [13–16]. Their expression is regulated also by the CRE sequence (http://natural.salk.edu/CREB). Thus, in a first series of experiments a possible influence of DHEAS on the expression of the tight-junction proteins claudin-3 and -5 was addressed at the mRNA/cDNA and protein levels. As shown by RT-PCR, treatment of TM4 cells for 48 h with 1 μM DHEAS clearly stimulated the expression of claudin-3- and claudin-5-specific mRNA/cDNA (S2A and S2B Fig) without having any effect on the expression of GAPDH-specific mRNA/cDNA, which served as a control (S2C Fig). While a basal quantity of claudin-5-specific mRNA/cDNA was also present in cells that were not exposed to DHEAS (S2B Fig), claudin-3-specific mRNA/cDNA was not detectable under basal conditions (S2A Fig).

**Fig 6:**
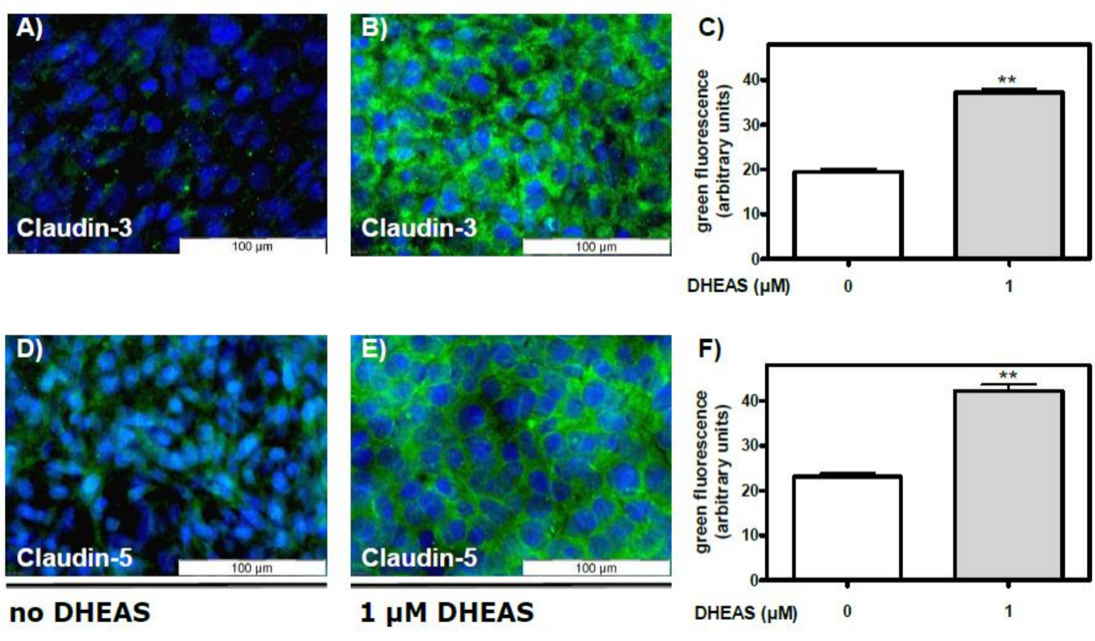
DHEAS-stimulated expression of claudin-3 and claudin-5 proteins as detected by immunofluorescence. TM4 Sertoli cells were cultured to 80% confluence and were further incubated for 2 days in the absence or presence of 1 μM DHEAS. Nuclei were labeled with DAPI and appear blue; the Alexa fluor 488-labeled secondary antibody shows the localization of claudin-3 or -5. (A and D) Fluorescence in the absence of DHEAS; (B and E) Fluorescence after exposure of cells to 1 μM DHEAS. (C) Quantification and statistical analysis of results shown in panels (A) and (B); (F) Quantification and statistical analysis of results shown in panels (D) and (E) (in each case n = 45; means ±SEM; **p≤0.01).

Expression of claudin-3-specific mRNA/cDNA correlated with the expression of claudin-3 protein as detected by immunofluorescence methods: whereas green fluorescence indicating the presence of claudin-3 protein was weak in cell cultures that had been grown in the absence of DHEAS for 48 h (Fig 6A), it was significantly greater in cultures that had been treated with 1 μM DHEAS under otherwise identical conditions (Fig 6B and 6C).

Similar effects of DHEAS were seen in immunofluorescence experiments measuring the expression of claudin-5 protein. Green fluorescence indicating the presence of claudin-5 protein was weak in control cultures (Fig 6D), and it was significantly increased in cultures that had been treated with 1 μM DHEAS under otherwise similar conditions (Fig 6E and 6F).

### Involvement of the classical AR receptor in the DHEAS-induced stimulation of claudin-3 and claudin-5 expression

**Fig 7:**
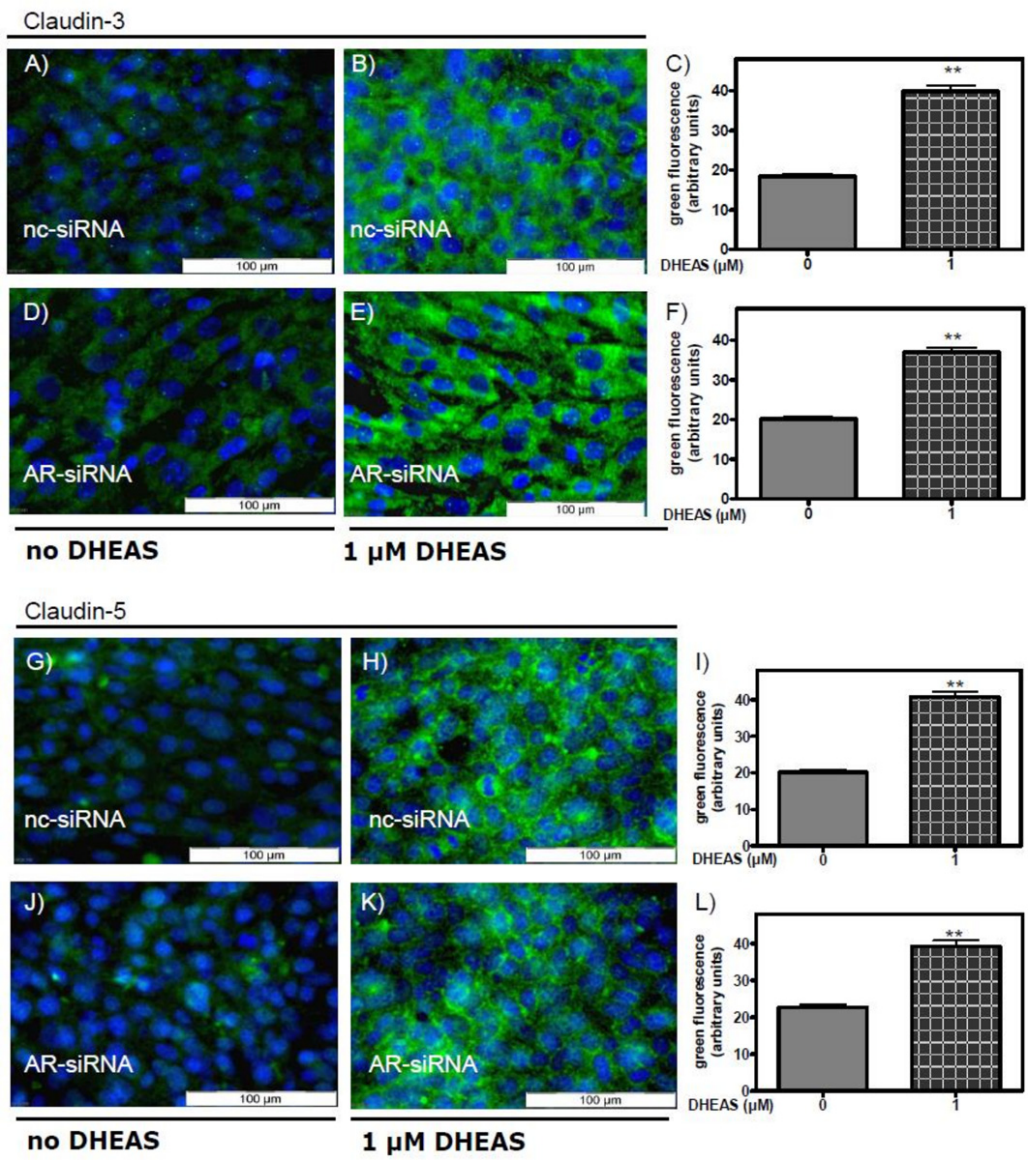
DHEAS-induced stimulation of claudin-3 and claudin-5 expression after silencing AR expression. The conditions are the same as in Fig 6, with the exception that the cells were incubated with either negative control siRNA (nc-siRNA) or with AR-specific siRNA (AR-siRNA) to prevent its expression before treatment with DHEAS. Nuclei of the cells were labeled with DAPI and appear blue. (A-F) Stimulation of claudin-3 expression by DHEAS after incubation of cells with either nc-siRNA (A, B, and C) or AR-siRNA (D, E and F). (G-L) Stimulation of claudin-5 expression by DHEAS after treatment of the cells with either nc-siRNA (G, H, and I) or with AR-siRNA (J, K, and L). For the statistics shown in C, F, I, and L: n = 45; means ±SEM; **p≤0.01.

The data shown in Fig 5 demonstrate that the effects of DHEAS on Erk1/2 phosphorylation do not depend on the presence of the classical AR. We next asked whether the AR is required for DHEAS stimulation of claudin-3 and claudin-5 expression. To address this possibility cells that had been treated with either nc-siRNA or AR-siRNA were incubated with 1 μM DHEAS under otherwise the same conditions as in Fig 6. Abrogation of AR expression had no effect on the DHEAS-induced stimulation of claudin-3 expression (Fig 7A–7F) or claudin-5 expression (Fig 7G–7L). Thus, although claudin expression has been shown to be regulated by androgens [14], the data shown in Fig 7 point towards an alternative signaling route that stimulates claudin-3 and claudin-5 expression by the interaction of DHEAS with a receptor that has yet to be identified.

### Stimulation of transepithelial resistance (TER) by DHEAS

**Fig 8:**
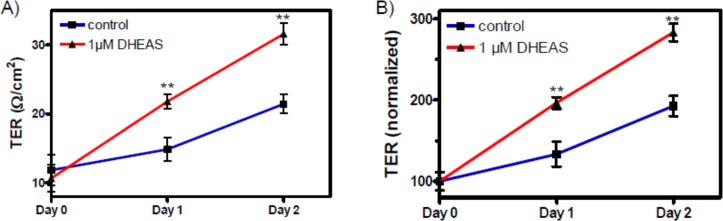
Tight junction formation between TM4 Sertoli cells in response to DHEAS. Cells were grown until they reached confluence. (A) Effects of incubation with 1 μM DHEAS for 1 or 2 days on TER (in Ω/cm^2^) across cell monolayers. Day 0 is the time point at which either DHEAS or vehicle alone were added to the cells. (B) Data from (A) normalized to the initial control value. For each data point: n = 4; means ± SEM; ** = p≤ 0.01.

The influence of DHEAS on the TJ between Sertoli cells was investigated by determining the transepithelial resistance (TER) across cell monolayers of TM4 cells. The exposure of TM4 cells to DHEAS for up to 48 h resulted in a significant increase in TER (Fig 8). Although this experiment cannot distinguish between effects associated with the expression of claudin-3 or claudin-5, it suggests that the increased TER caused by DHEAS is due to the increased expression and participation of either or both of the two claudins in TJ formation between neighboring TM4 cells. Nevertheless, by taking into consideration that claudin-3 seems to be evenly distributed over the entire cell, whereas claudin-5 is clearly also found between neighboring cells (Fig 6E), one might expect that it is rather claudin-5 that participates in TJ formation and causes an increase in TER than claudin-3.

### Involvement of Gnα11 in DHEAS-induced signaling

Some steroid hormones do not elicit their actions solely through nuclear receptors; they additionally mediate rapid effects through their interactions with G-protein-coupled receptors (GPCRs) [25, 32–38].

**Fig 9:**
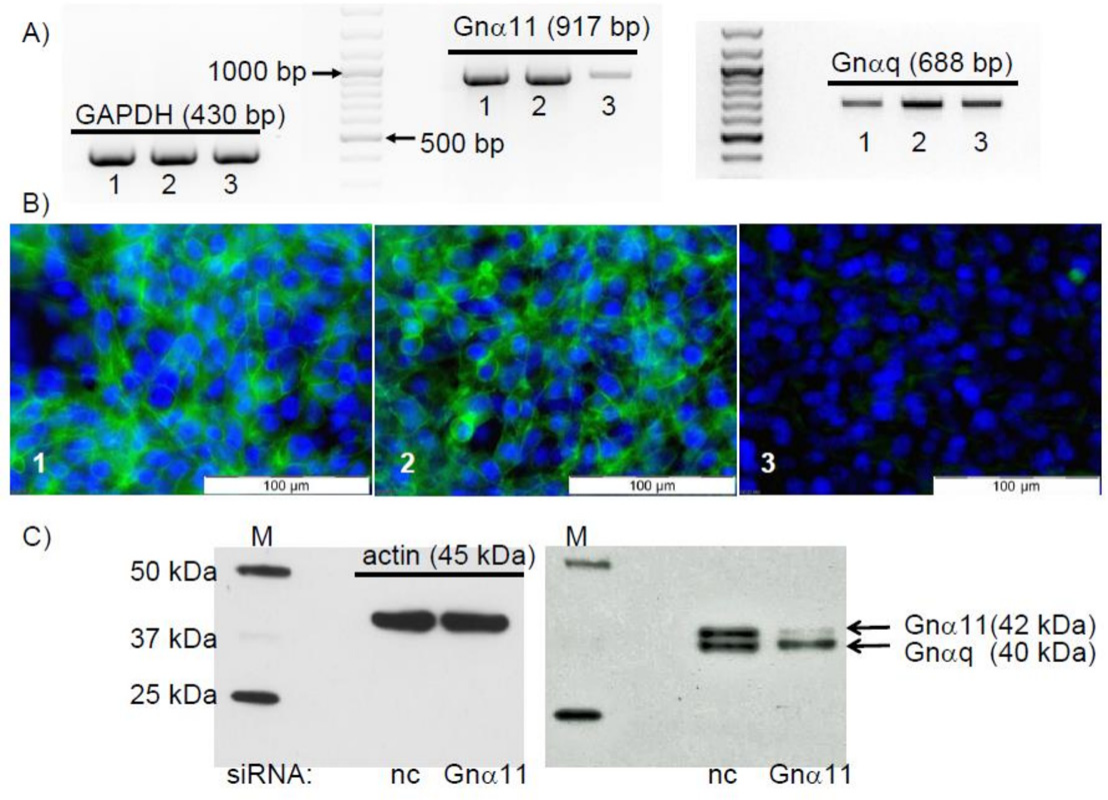
Silencing expression of Gnα11 by means of siRNA. Cells were treated for 3 days with OptiMem plus Lipofectamine RNAiMAX alone (1) or OptiMem plus Lipofectamine RNAiMAX plus the siRNA negative control (2) or OptiMem plus Lipofectamine RNAiMAX plus the Gnα11-specific siRNA (3). Cells were then used to either isolate mRNA for RT-PCR, for immunofluorescence or for western blot experiments. (A) RT-PCR for the detection of GAPDH-, Gnα11- or Gnαq-specific mRNA. Effects on GAPDH mRNA/cDNA (430 bp) are shown on the left, results for Gnα11-specific mRNA/cDNA (917 bp) are shown in the center, and effects on Gnαq (688 bp) are shown on the right. (B) Detection of Gnα11 by immunofluorescence. The green fluorescence indicates Gnα11, and the blue refers to DAPI-stained nuclei. In both western blotting (A) and immunofluorescence (B) experiments, treatment of cells with Gnα11-specific siRNA (panel labeled “3”) abrogates Gnα11 expression. (C) Results of western blotting using an antibody against Gnα11 on lysates from cells treated with either nc-siRNA or Gnα11-siRNA. Effects on expression of actin (left panel) and Gnα11 (right panel, upper band, 42 kDa). The lower band also recognized by the antibody is Gnαq (40 kDa). All results shown are representative of n = 3 similar experiments.

The RT-PCR results shown in Fig 9 demonstrate the presence of Gnα11-specific mRNA/cDNA in the Sertoli cell line TM4. In order to investigate a possible involvement of this protein in the observed signaling events, siRNA experiments were carried out to suppress its expression. Transfection of TM4 cells with the Gnα11-specific siRNA oligo pair led to a significant reduction of the expression of Gnα11-specific mRNA/cDNA (Fig 9A, middle panel, lane 3). Under the same conditions, expression of GAPDH-specific or Gnαq-specific mRNA/cDNA were not affected (Fig 9A, left and right), indicating that the reduction of the expression of Gnα11-specific mRNA/cDNA was specifically induced by the Gnα11-specific siRNA oligo pair used. Neither the expression of Gnα11-specific, Gnαq-specific or GAPDH-specific mRNA/cDNA was affected when OptiMem plus Lipofectamine RNAiMAX alone (Fig 9A, lanes 1) or OptiMem plus Lipofectamine RNAiMAX plus the siRNA negative control (Fig 9A, lanes 2) were used instead. Although the amount of Gnα11-specific mRNA/cDNA is highly reduced after treatment of the cells with Gnα11-specific siRNA, the expression of the targeted protein might not be as rapidly affected. We therefore addressed by immunofluorescence whether the Gnα11 protein was still present in the cells despite the reduction of Gnα11-specific mRNA/cDNA by siRNA. Although green fluorescence, indicating the expression of the Gnα11 protein, was visible in every TM4 cell treated either with OptiMem plus Lipofectamine RNAiMAX alone (Fig 9B1) or with OptiMem plus Lipofectamine RNAiMAX plus the siRNA negative control (Fig 9B2), it was almost entirely absent after treatment of the cells with Gnα11-siRNA to prevent expression of Gnα11-specific mRNA (Fig 9B3). This finding was further examined in the western blot experiments shown in Fig 9C. Treatment of cells with nc-siRNA or Gnα11-siRNA did not affect the expression of actin, indicating that a general effect on protein expression is unlikely (Fig 9C, left panel). Similarly, treatment of the cells with nc-siRNA did not affect the expression of Gnα11 (Fig 9C, right panel). Application of Gnα11-siRNA, however, almost completely abolished the expression of Gnα11 protein (Fig 9C, right panel, upper band). The lower band that is recognized by the antibody is Gnαq (40 kDA; accession #: AAH11169.1) [39], which shares an 82% identity with Gnα11 (accession #: AAB36839.1) in the area of the epitope chosen by the producer of the antibody (amino acids 3–39 within the N-terminus). Under the denaturing conditions used for western blotting this antibody recognizes the similar epitopes of Gnα11 and Gnαq (Fig 9C), but under the conditions of the immunofluorescence experiments (Fig 9B) the antibody displays a higher specificity for the original target Gnα11, and cross-reactivity with similar epitopes remains at a minimum. In addition, the results also underline the high specificity of the siRNA, which precisely targeted the Gnα11 mRNA and not mRNAs of the related Gnαq.

**Fig 10:**
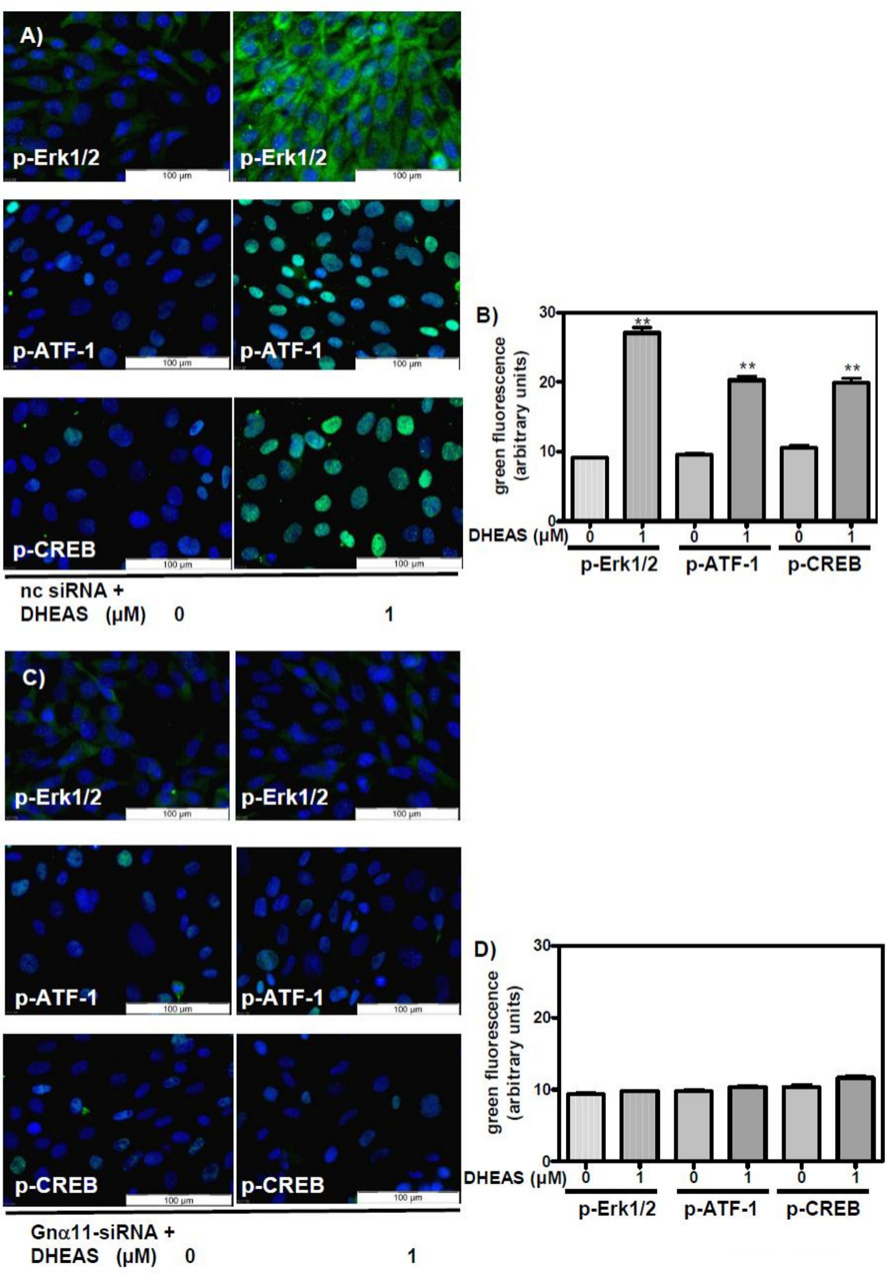
Detection of phospho-Erk1/2, phospho-CREB, and phospho-ATF-1 by immunofluorescence after silencing Gnα11 expression by siRNA. In all photomicrographs the green fluorescence indicates activated Erk1/2 or transcription factors CREB and ATF-1, and the blue fluorescence shows DAPI-stained nuclei. (A) All cells were treated with OptiMem plus Lipofectamine RNAiMAX and negative control siRNA (nc-siRNA). Cells in the leftmost three panels were treated with vehicle only (no DHEAS). The cells of the right-hand three panels were treated with 1 μM DHEAS. (B) Quantification and statistical analysis of results shown in panels in (A) (n = 90; means ±SEM; **p≤0.01). (C) All cells were treated with OptiMem, Lipofectamine RNAiMAX, and Gnα11-specific siRNA (Gnα11-siRNA). Cells in the leftmost three panels were treated with vehicle only (no DHEAS), and cells in the right-hand three panels were treated with 1 μM DHEAS. (D) Quantification and statistical analysis of results shown in panels in (C) (n = 90; means ±SEM).

Treatment of TM4 cells with negative control siRNA (nc-siRNA) did not affect phosphorylation of Erk1/2, CREB, or ATF-1 by 1 μM DHEAS (Fig 10A and 10B). In cells that were exposed to Gnα11-specific siRNA, however, the stimulatory effects of DHEAS on Erk1/2, CREB, or ATF-1 were abrogated: fluorescence corresponding to p-Erk1/2, p-CREB or p-ATF-1 was at the same level as the fluorescence measured in the absence of the steroid (Fig 10C and 10D).

**Fig 11:**
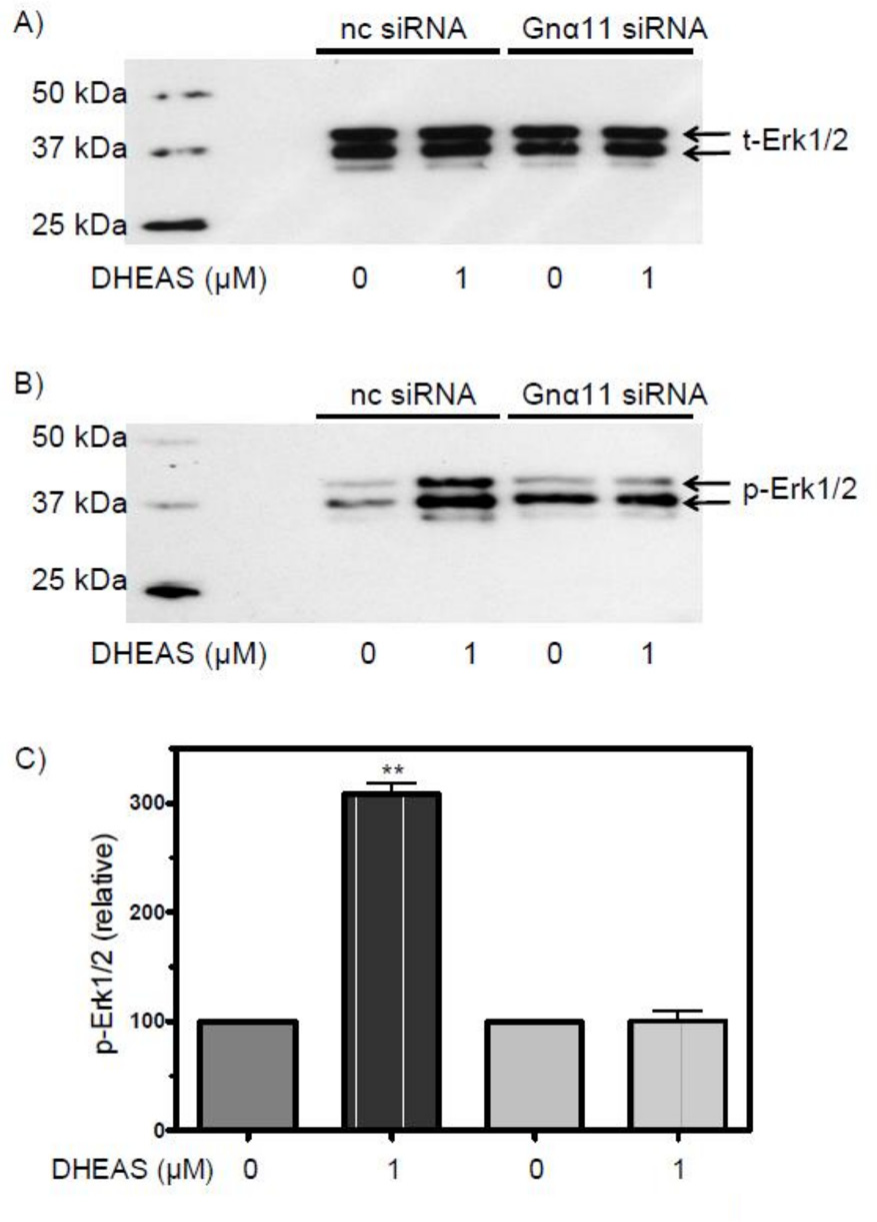
Western blot analysis of phospho-Erk1/2 after silencing Gnα11 expression by siRNA. (A) Blots showing the amount of total Erk1/2 and (B) phospho-Erk1/2 in cells pre-treated with OptiMem plus Lipofectamine RNAiMAX and negative control siRNA (nc-siRNA) or Gnα11-specific siRNA and incubated subsequently in the presence or absence of 1 μM DHEAS. (C) For statistical analysis, data were corrected for the amount of total Erk1/2 as shown in panel (A) (n = 4; means ± SEM; * = p≤ 0.01).

The western blotting results shown in Fig 11 confirm these immunofluorescence experiments. Silencing Gnα11 expression by transfecting the TM4 cells with Gnα11-specific siRNA blocked the DHEAS-induced activation of Erk1/2 (Fig 11). Treatment of the cells with nc-siRNA did not affect p-Erk1/2 formation (Fig 11), which was similar to that of cells not exposed to siRNA (Fig 2).

**Fig 12:**
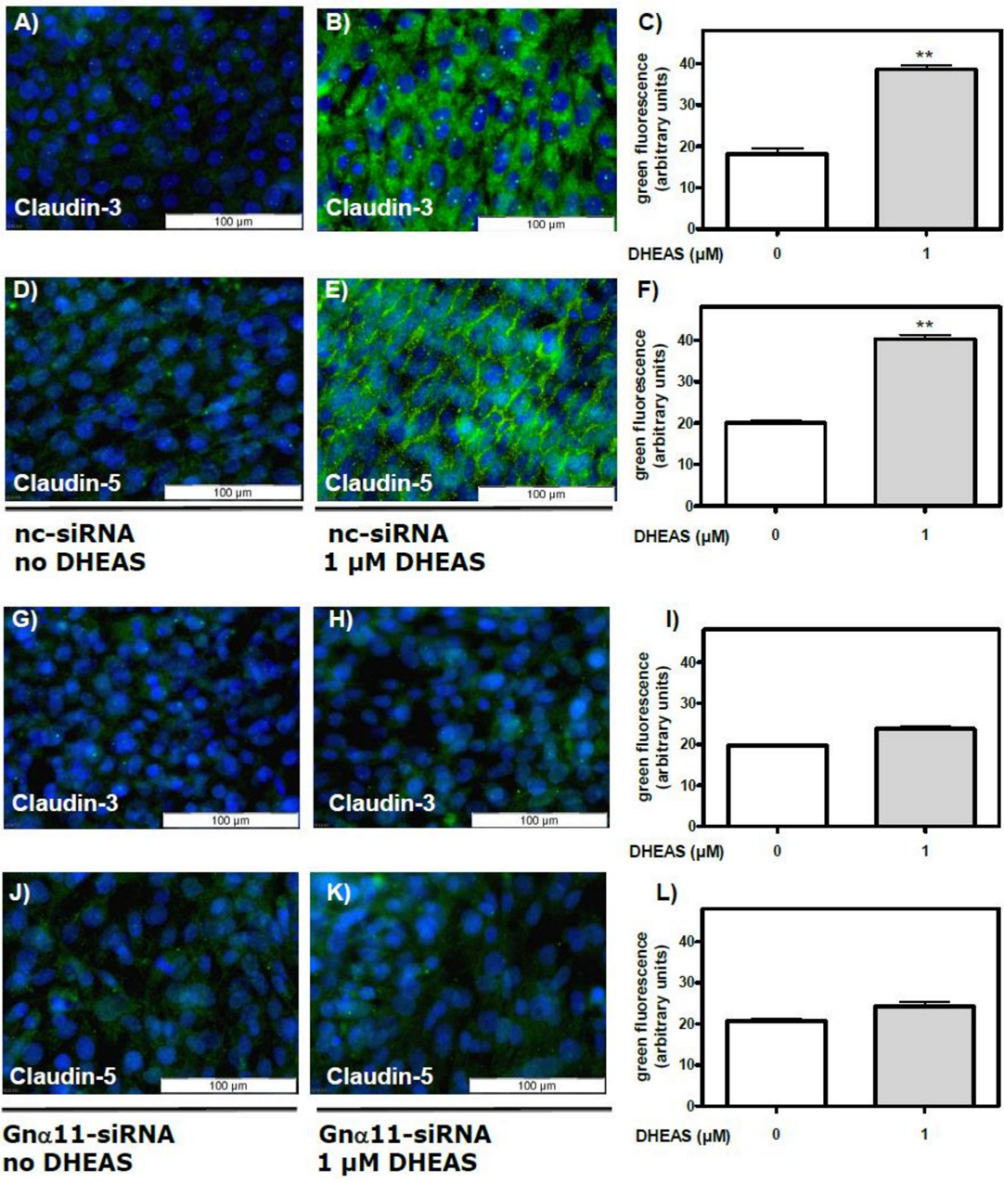
DHEAS effects on claudin-3 and claudin-5 expression after silencing Gnα11 expression by siRNA. Cells treated for 3 days with nc-siRNA or Gnα11 siRNA were grown for a further 48 h in the presence or absence of 1 μM DHEAS. Then they were subjected to a fixation/immunostaining procedure to detect claudin-3 or -5 by immunofluorescence, as in experiments depicted in Fig 9. In all panels green fluorescence indicates claudin-3 or -5 proteins and blue indicates DAPI-stained nuclei. Effects of treatment with nc-siRNA on the stimulation of claudin-3 (panels A through C) or claudin-5 (D through F) expression by DHEAS. Effects of treatment with Gnα11 siRNA on the stimulation of claudin-3 (G through I) or claudin-5 (J through L) expression by DHEAS. (For the statistical analysis shown in C, F, I, and L; n = 90; means ±SEM; **p≤0.01).

**Fig 13:**
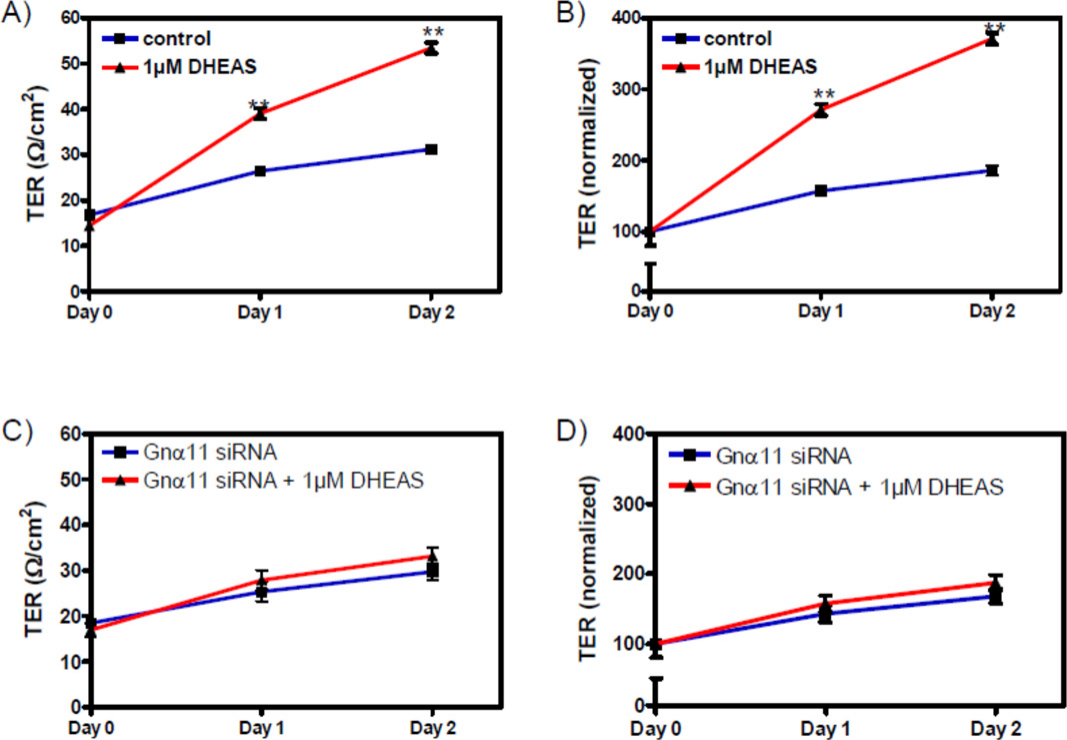
Effect of DHEAS on tight junction formation after suppressing Gnα11 expression by siRNA. Cells were pre-treated with negative control siRNA (nc-siRNA) or siRNA against Gnα11 (Gnα11 siRNA) for 3 days. TER measurements (given in Ω/cm^2^) were carried out as described under “Methods” and in the legend to Fig 8. (A and B) Effects of DHEAS on TER in cells pre-treated with nc-siRNA, similar to results shown in Fig 8. (C and D) Effects of DHEAS on TER in cells pre-treated with Gnα11 siRNA. Results shown in (B) and (D) are normalized values of (A) and (C), respectively. For each data point: n = 4; means ± SEM; ** = p≤ 0.01.

The results presented in Fig 12 show that the stimulatory effect of DHEAS on claudin-3 (Fig 12G–12I) or claudin-5 expression (Fig 12J–12L) was suppressed when the expression of Gnα11 was reduced by means of siRNA. At the same time, nc-siRNA did not affect the expression of either claudin-3 (Fig 12A–12C) or claudin-5 (Fig 12D–12F). Further experiments showed that Gnα11-specific siRNA abrogated the DHEAS-induced increase in TER (Fig 13C and 13D), indicating that TJ formation between adjacent Sertoli cells is greatly impaired when the DHEAS-induced signaling cascade is disrupted.

## Discussion

The results of the present investigation are consistent with the idea that DHEAS might not be simply an inactive proandrogen reservoir or merely a waste product of steroid hormone metabolism but rather may act as a hormone on its own, which could be of physiological significance for male fertility. This proposal is based on the fact that physiological concentrations of DHEAS induce a signaling cascade in the Sertoli cell line TM4 that resembles the non-classical signaling pathway of steroid hormones, which considerably differs from their classical actions involving intracellular receptors that essentially function as ligand-activated transcription factors [40]. Thus, nanomolar and low micromolar concentrations of DHEAS trigger the activation of Erk1/2, CREB, and ATF-1, as demonstrated in the current investigation by immunofluorescence experiments and western blots (Figs 1 and 2). The identification of these signaling events is consistent with previous investigations showing that sulfated steroids can act autonomously as steroid hormones [4–7, 38]. Accordingly, use of the steroid sulfatase inhibitor STX64 (Fig 3) or abrogation of AR expression (Fig 5) failed to prevent DHEAS-induced phosphorylation of Erk1/2 (Fig 5), and DHEA in place of DHEAS failed to stimulate phosphorylation of the same kinase (Fig 4). Taken together, these results indicate that conversion of DHEAS to DHEA or testosterone is not required to induce the signaling events examined here.

The signaling cascade revealed here in a Sertoli cell line may be of physiological significance. Activation of Erk1/2 (and also of other mitogen-activated protein kinases) is essential for spermatogenesis and maturation of spermatogonia to haploid spermatozoa [41–44]. In Sertoli cells, activated Erk1/2 stimulates cell proliferation [45, 46]. Activation of the transcription factors CREB and ATF-1 is also of physiological significance for male fertility. Both proteins are members of the bZIP superfamily of transcription factors and stimulate transcription when phosphorylated either at Ser133 (CREB) or at Ser63 (ATF-1) residues localized within a conserved region termed the phosphorylation box [47]. Transcription factors like CREB or ATF-1 that bind to CRE sequences within certain promoters induce the transcription of a great variety of genes, and CREB/CRE-inducible transcription is essential for the survival of spermatocytes and the production of mature spermatozoa [48]. The amount of phospho-CREB varies during the spermatogenic cycle [49], which would be consistent with it being directly involved in the differentiation process of germ cells. In Sertoli cells CREB activation is proposed to be involved in the regulation of the spermatogenic cycle [50]. In addition, activation of CREB in Sertoli cells via the non-classical testosterone signaling pathway (involving Erk1/2 activation) is essential for spermatogenesis and maintenance of male fertility [51, 52]. These observations reflect the interplay between Sertoli and spermatogenic cells. It is yet unknown, however, whether and how activated CREB (or ATF-1) might directly influence the physiology of Sertoli cells, although its involvement in the regulation of Sertoli cell function has already been proposed but not further elaborated by others [53].

To address the possibility of direct effects of DHEAS and CREB/ATF-1 activation on Sertoli cells we investigated the effects of the steroid on the expression of claudins. The rationale for this approach is based on the fact that expression of several claudins is regulated by CRE sequences and that claudins, as constituents of TJ, participate considerably in the formation and maintenance of the BTB [54].

The BTB is one of the tightest blood-tissue barriers in mammals and separates the seminiferous epithelium into basal and adluminal compartments. The main function of the BTB is the formation of an immunological barrier in order to protect the meiotic and post-meiotic stages of the germ cells from cells of the immune system. Disturbance of the integrity of the BTB causes infertility [55].

Formation and maintenance of the BTB is mainly defined by the formation of TJ between neighboring Sertoli cells [55, 56]. TJ, in turn, are formed by the interactions of claudins, which interact with signalling proteins and proteins of the cytoskeleton on the cytosolic side of the membrane [54]. Various forms of claudins are expressed in rodent testes. The increased expression of claudin-3 and -5 at stage VIII of spermatogenesis and their appearance at the BTB at postnatal day 20 [14, 15, 57] suggests their involvement in the formation and dynamics of this barrier [15]. Their homophilic interactions with their respective isoforms and heterophilic interactions between them considerably influences the formation of TJ and the transepithelial resistance of cell cultures that express both proteins [58, 59].

Our results show that DHEAS stimulates the expression of claudin-3 and -5 at the mRNA/cDNA and protein levels (Fig 6 and S2 Fig) and increases the TER (Fig 8), consistent with the formation of TJ between adjacent TM4 cells. This latter effect can probably be attributed to claudin-5, which is also found between neighboring cells (Fig 6E). Claudin-3, which is known to be associated with newly formed tight junctions only [14, 60], is evenly distributed over the entire cell and therefore probably does not contribute to the increased TER. Although claudin expression has been shown to be regulated by androgens [14], the classical AR does not seem to be involved in the signaling events that lead to increased expression of claudin-3 and -5, since abrogation of its expression does not affect the stimulatory effect of DHEAS (Fig 7). These findings, together with the data summarized in Figs 3–5, indicate that conversion of DHEAS to DHEA or testosterone is not required for the sequence of events described here. Thus, one has to assume that a specific DHEAS receptor might be directly involved in the DHEAS-induced signaling. The question that remains, however, is what kind of receptor is it?

G-protein coupled receptors (GPCRs) have been shown to trigger activation of Erk1/2 in numerous signaling cascades [61–63]. In addition, various non-classical actions of steroid hormones are mediated through GPCRs [32–35]. In the mast cell line RBH-2H3 the Gq/11 protein was shown to be involved in DHEAS actions [64]. Based on these facts and because Gnα11, as member of the Gq class of heterotrimeric G proteins [63] was found to mediate DHEAS signaling in the spermatogenic cell line GC-2 [38], we investigated a possible involvement of this protein in the DHEAS-induced signaling described here. Immunofluorescence and western blot experiments demonstrated the importance of this protein, since silencing its expression by means of siRNA completely abolishes the DHEAS-induced activation of either Erk1/2 or the transcription factors CREB and ATF-1 (Figs 10 and 11). Under these conditions, in the presence of DHEAS the expression of TJ proteins claudin-3 and -5 (Fig 12H and 12K) and the TER (Fig 13C and 13D), indicative of TJ formation, remain at the levels observed in the control cultures grown in the absence of DHEAS, indicating the involvement of Gnα11 and a membrane-bound GPCR in the stimulation of these responses.

Although this receptor has yet to be identified, the results of the current investigation (summarized in S3 Fig) clearly point out that DHEAS acts as a hormone in its own right. Taking into consideration that DHEAS is produced in the gonads of humans [65] or rodents [66], one might expect similar effects of the steroid *in vivo*. Thus, by influencing the maintenance and dynamics of the BTB, DHEAS might contribute to the safeguarding of male fertility. Furthermore, since DHEAS is also produced in brain or adrenal cortex and claudin-3 or -5 are also constituents of various other blood-tissue barriers, it would not be unexpected if it were revealed that DHEAS influences the dynamics of these tissue-blood barriers as well. Thus, the extension of this investigation to other tissues and cell types might help to define new actions of DHEAS and establish its function as an essential steroid hormone in mammalian physiology.

## Supporting Information

S1 Fig Identification of Sertoli cell-specific markers in TM4 cells: RT-PCR demonstrates the expression of mRNA/cDNA for the Sertoli cell-specific markers Sox9 (regulates the differentiation of Sertoli cells in the testis), ABP (androgen binding protein; a functional marker of Sertoli cells), and Dhh (Desert Hedgehog; regulates the male germ line in RT-PCR). Taking these data together with the fact that TM4 also express AR (androgen receptors), one can assume that TM4 cells constitute a reliable model for studying Sertoli cell properties.
(TIF)

S2 Fig Stimulation of expression of claudin-3- and claudin-5-specific mRNA/cDNA detected by RT-PCR: TM4 cells were exposed to 1 μM DHEAS for 48 h. Cells were then harvested for mRNA extraction, reverse transcription and PCR as detailed in “Methods”. (A) DHEAS-stimulated expression of claudin-3-specific mRNA/cDNA (results of two duplicate experiments are shown). (B) DHEAS-stimulated expression of specific mRNA/cDNA for claudin-5. (C) GAPDH-specific mRNA/cDNA expression in the same experiments.
(TIF)

S3 Fig Summary of the DHEAS-induced signaling events induced in the Sertoli cell line TM4. Interaction of DHEAS with a still undefined GPCR stimulates a signaling cascade responsible for the non-classical actions of steroid hormones. This signaling cascade is mediated by Gnα11, which leads to Erk1/2 activation and to stimulation of the transcription factors CREB and ATF-1. Activated CREB and possibly ATF-1 stimulate the transcription of claudin-3- and claudin-5-specific mRNAs that are under the control of CRE sequences. As a result, claudin-3 and -5 protein expression and TJ formation between adjacent Sertoli cells are significantly increased.
(TIF)

S1 Table The table contains all raw data used in the statistical analyis. (DOC)
