# Correction: Dehydroepiandrosterone Sulfate Stimulates Expression of Blood-Testis-Barrier Proteins Claudin-3 and -5 and Tight Junction Formation via a Gnα11-Coupled Receptor in Sertoli Cells

**DOI:** 10.1371/journal.pone.0153293

**Published:** 2016-04-05

**Authors:** The PLOS ONE Staff

The following information is missing from the Funding section: This study was supported by a grant from the Deutsche Forschungsgemeinschaft (SCHE 307/7-1) to GSB. The publisher apologies for this error.
