# Supplementary figures and images for: Dehydroepiandrosterone Sulfate Stimulates Expression of Blood-Testis-Barrier Proteins Claudin-3 and -5 and Tight Junction Formation via a Gnα11-Coupled Receptor in Sertoli Cells
Source: PLoS One. 2016 Mar 3;11(3):e0150143. doi: 10.1371/journal.pone.0150143 (PMC4777551; doi:10.1371/journal.pone.0150143)

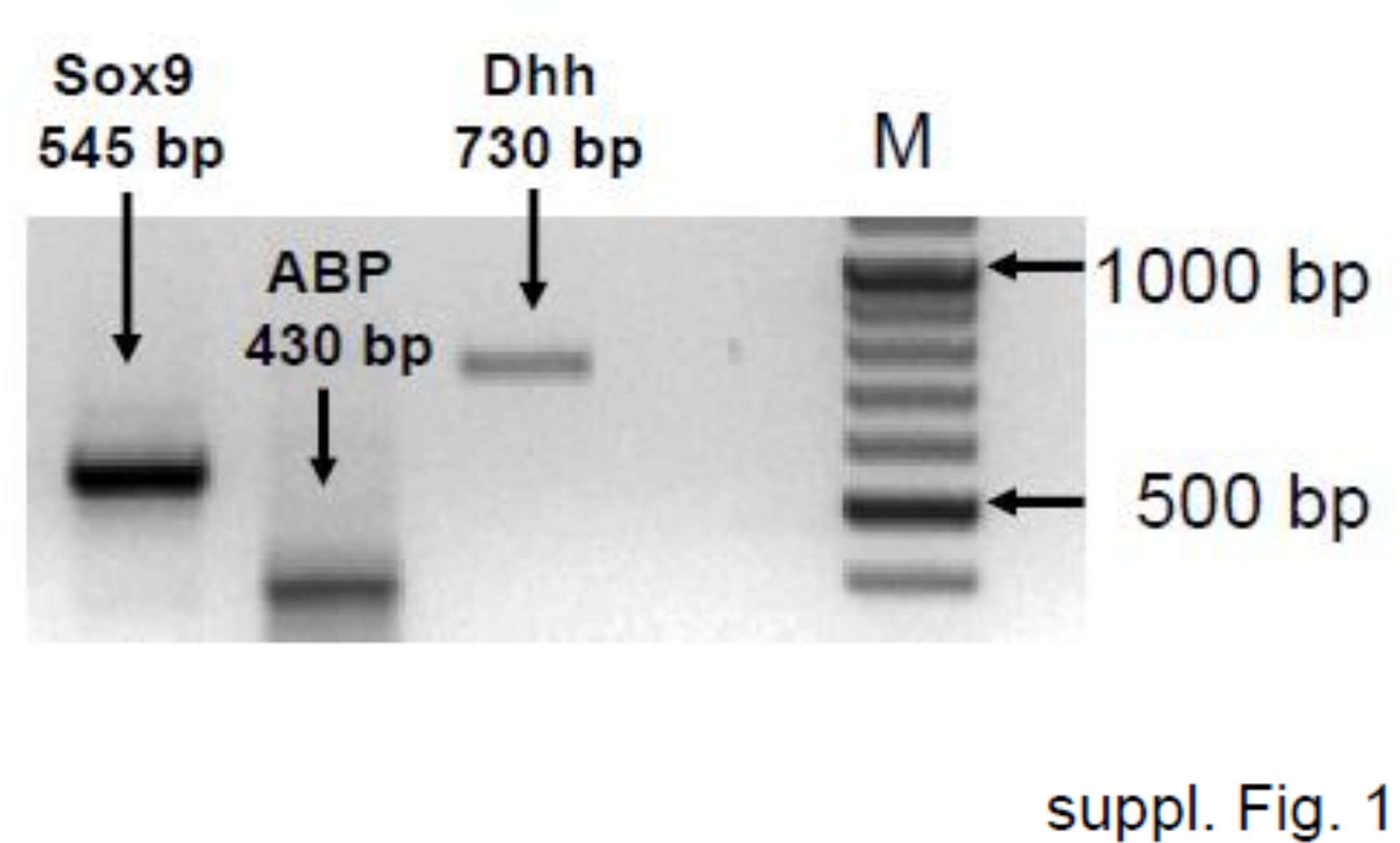

Supplement: S1 Fig — Taking these data together with the fact that TM4 also express AR (androgen receptors), one can assume that TM4 cells constitute a reliable model for studying Sertoli cell properties. (TIF) [file pone.0150143.s001.tif]

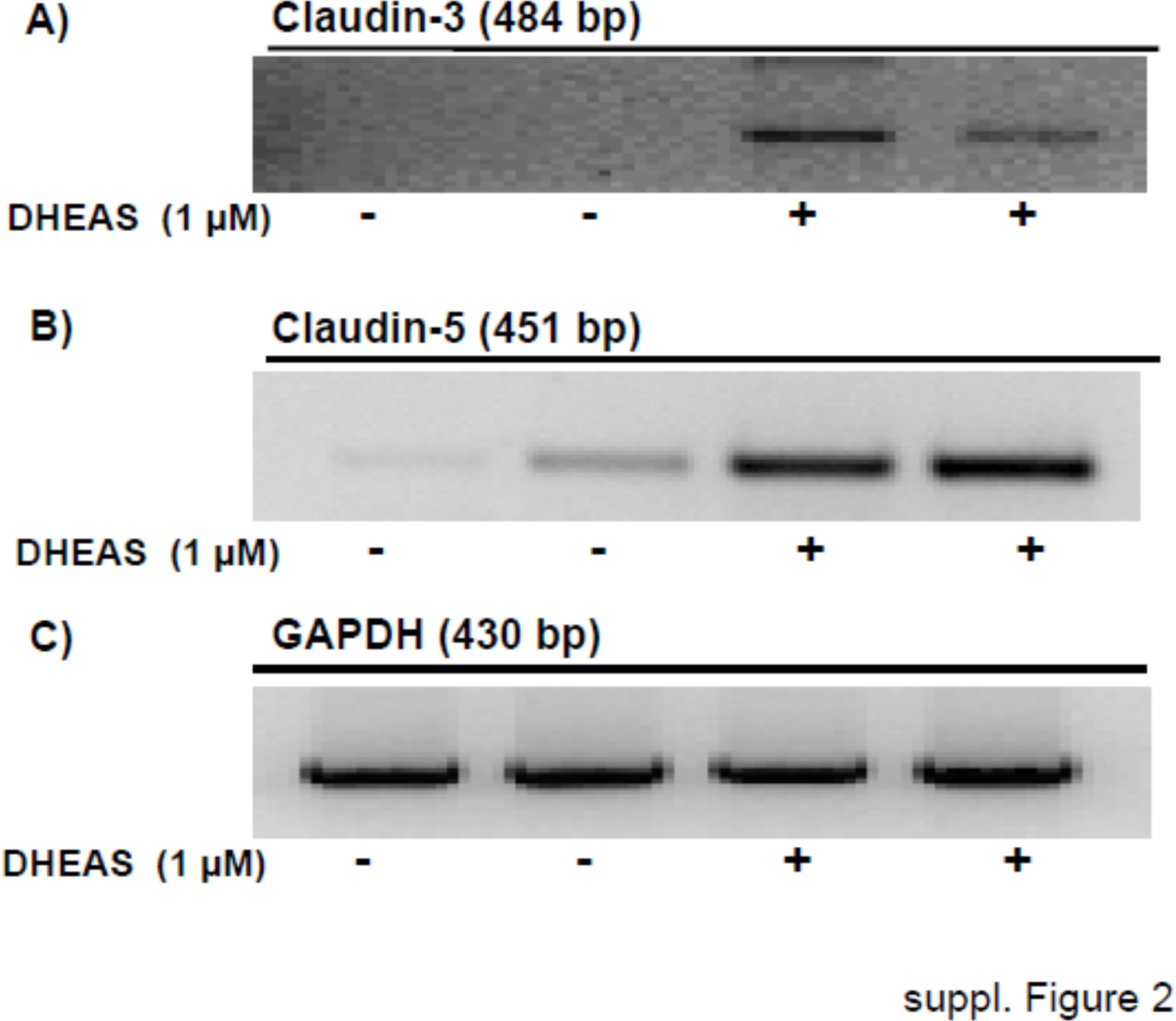

Supplement: S2 Fig — Cells were then harvested for mRNA extraction, reverse transcription and PCR as detailed in “Methods”. (A) DHEAS-stimulated expression of claudin-3-specific mRNA/cDNA (results of two duplicate experiments are shown). (B) DHEAS-stimulated expression of specific mRNA/cDNA for claudin-5. (C) GAPDH-specific mRNA/cDNA expression in the same experiments. (TIF) [file pone.0150143.s002.tif]

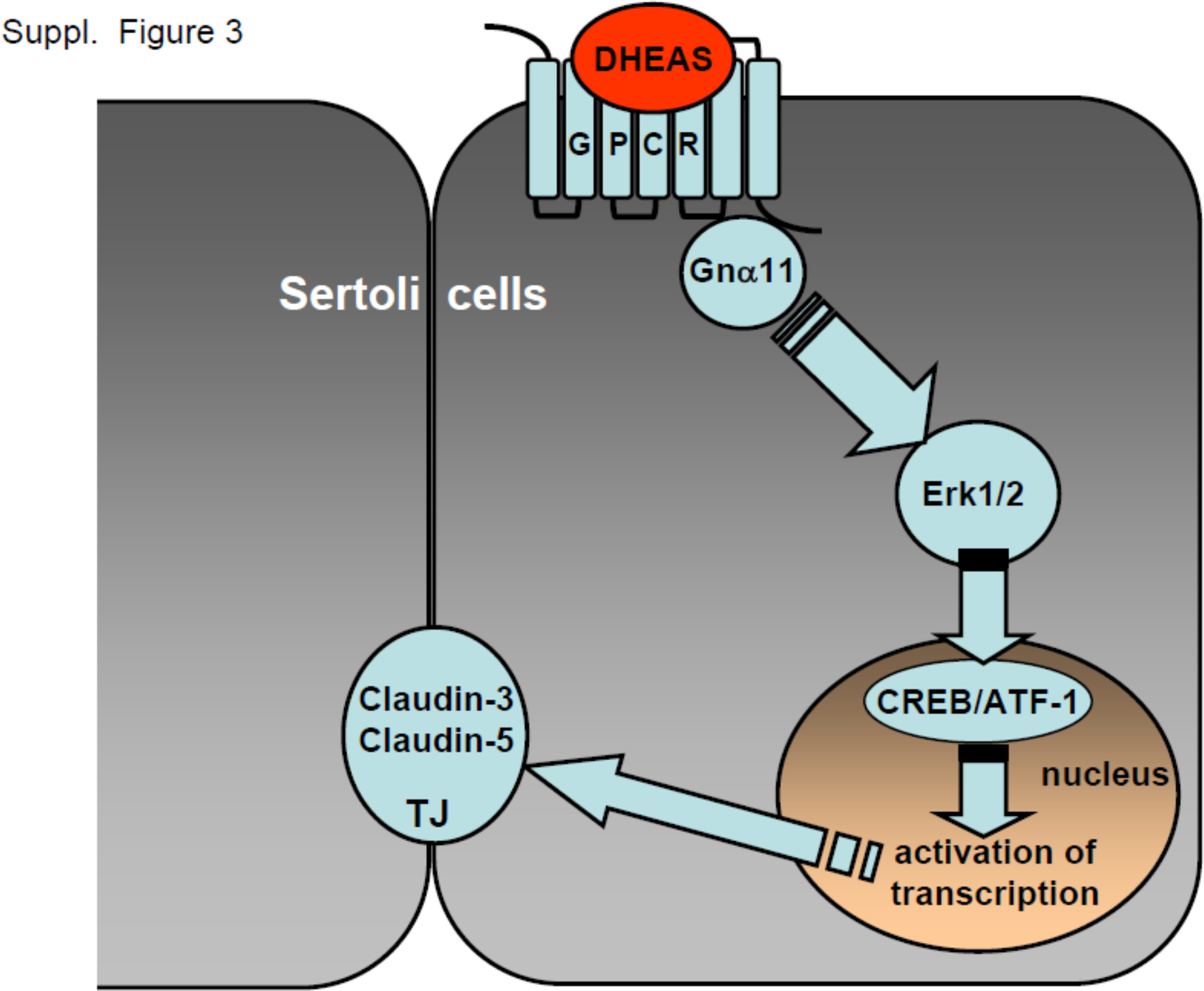

Supplement: S3 Fig — Interaction of DHEAS with a still undefined GPCR stimulates a signaling cascade responsible for the non-classical actions of steroid hormones. This signaling cascade is mediated by Gnα11, which leads to Erk1/2 activation and to stimulation of the transcription factors CREB and ATF-1. Activated CREB and possibly ATF-1 stimulate the transcription of claudin-3- and claudin-5-specific mRNAs that are under the control of CRE sequences. As a result, claudin-3 and -5 protein expression and TJ formation between adjacent Sertoli cells are significantly increased. (TIF) [file pone.0150143.s003.tif]
