# Supplementary material for: Dehydroepiandrosterone Sulfate Stimulates Expression of Blood-Testis-Barrier Proteins Claudin-3 and -5 and Tight Junction Formation via a Gnα11-Coupled Receptor in Sertoli Cells
Source: PLoS One. 2016 Mar 3;11(3):e0150143. doi: 10.1371/journal.pone.0150143 (PMC4777551; doi:10.1371/journal.pone.0150143)
Supplement: S1 Table — (DOC) [file pone.0150143.s004.doc]

**S4 Table:** Raw Data

| **Figure 1C** | | **Figure 1F** | | **Figure 1I** | |  |  |
| --- | --- | --- | --- | --- | --- | --- | --- |
| **0 µM** | **1 µM** | **0 µM** | **1 µM** | **0 µM** | **1 µM** |  |  |
| 9,579 | 24,645 | 18,211 | 20,123 | 15,534 | 15,675 |  |  |
| 11,150 | 22,096 | 11,237 | 31,696 | 17,193 | 29,254 |  |  |
| 10,974 | 27,955 | 9,318 | 30,736 | 13,917 | 24,766 |  |  |
| 14,665 | 33,362 | 14,345 | 25,641 | 12,084 | 19,386 |  |  |
| 11,075 | 28,459 | 11,240 | 26,411 | 15,867 | 23,664 |  |  |
| 9,184 | 37,666 | 13,792 | 23,487 | 12,260 | 36,032 |  |  |
| 11,809 | 30,898 | 14,017 | 27,564 | 16,280 | 52,890 |  |  |
| 9,704 | 24,549 | 10,535 | 32,469 | 15,975 | 29,568 |  |  |
| 11,200 | 22,276 | 12,420 | 29,970 | 14,860 | 31,009 |  |  |
| 12,435 | 27,285 | 15,826 | 22,757 | 12,628 | 24,376 |  |  |
| 11,342 | 26,517 | 13,966 | 23,989 | 12,567 | 29,280 |  |  |
| 9,386 | 28,816 | 9,023 | 27,717 | 11,798 | 29,793 |  |  |
| 13,077 | 19,272 | 9,319 | 20,093 | 10,915 | 17,335 |  |  |
| 18,072 | 17,487 | 14,240 | 23,081 | 9,689 | 21,043 |  |  |
| 19,889 | 22,121 | 23,858 | 20,822 | 9,994 | 21,389 |  |  |
| 11,585 | 21,805 | 15,080 | 20,549 | 11,017 | 25,989 |  |  |
| 20,652 | 26,534 | 12,424 | 19,947 | 17,423 | 29,956 |  |  |
| 15,575 | 18,147 | 9,206 | 21,795 | 11,516 | 30,321 |  |  |
| 8,580 | 25,065 | 8,079 | 23,298 | 11,936 | 25,477 |  |  |
| 8,815 | 27,206 | 8,958 | 21,830 | 14,051 | 19,886 |  |  |
| 8,898 | 41,079 | 14,500 | 26,823 | 9,953 | 31,675 |  |  |
| 9,216 | 20,632 | 14,822 | 16,866 | 19,036 | 26,075 |  |  |
| 10,840 | 21,718 | 9,602 | 21,172 | 14,308 | 23,701 |  |  |
| 9,576 | 28,539 | 9,476 | 25,346 | 13,805 | 20,069 |  |  |
| 9,854 | 22,962 | 15,248 | 19,746 | 11,923 | 20,385 |  |  |
| 9,367 | 36,068 | 10,703 | 24,823 | 10,254 | 39,118 |  |  |
| 9,306 | 34,107 | 11,857 | 26,780 | 19,723 | 31,584 |  |  |
| 9,943 | 22,589 | 12,833 | 16,200 | 15,183 | 17,935 |  |  |
| 12,439 | 27,031 | 8,603 | 28,969 | 10,269 | 19,888 |  |  |
| 12,428 | 22,727 | 11,102 | 21,572 | 13,923 | 20,979 |  |  |
| 8,531 | 25,500 | 13,318 | 19,266 | 13,118 | 16,863 |  |  |
| 12,854 | 32,065 | 9,664 | 18,088 | 13,219 | 20,119 |  |  |
| 13,961 | 27,501 | 10,039 | 26,108 | 14,273 | 35,872 |  |  |
| 11,946 | 29,637 | 11,085 | 21,737 | 10,685 | 45,346 |  |  |
| 9,931 | 28,277 | 11,290 | 25,886 | 9,562 | 24,000 |  |  |
| 12,567 | 26,689 | 8,260 | 21,088 | 9,257 | 18,030 |  |  |
| 11,176 | 30,122 | 9,570 | 21,705 | 8,974 | 21,763 |  |  |
| 9,325 | 22,282 | 8,629 | 22,708 | 11,504 | 25,987 |  |  |
| 17,283 | 26,472 | 13,296 | 39,175 | 9,225 | 19,408 |  |  |
| 13,351 | 29,138 | 10,244 | 27,359 | 11,364 | 30,326 |  |  |
| 8,357 | 24,987 | 10,234 | 21,382 | 9,459 | 14,182 |  |  |
| 11,097 | 28,466 | 13,535 | 25,559 | 12,011 | 34,711 |  |  |
| 15,231 | 19,159 | 10,790 | 25,683 | 12,616 | 38,391 |  |  |
| 8,307 | 24,716 | 9,426 | 28,186 | 11,107 | 20,074 |  |  |
| 11,137 | 25,056 | 8,670 | 29,166 | 11,711 | 18,398 |  |  |
| 9,947 | 18,901 | 13,036 | 20,530 | 12,294 | 28,354 |  |  |
| 12,609 | 29,975 | 12,155 | 22,052 | 9,986 | 23,290 |  |  |
| 9,558 | 26,710 | 8,311 | 29,413 | 9,804 | 22,041 |  |  |
| 10,665 | 33,001 | 8,919 | 36,027 | 9,627 | 27,212 |  |  |
| 12,903 | 21,952 | 9,610 | 21,437 | 15,105 | 24,251 |  |  |
| 10,128 | 16,569 | 9,222 | 19,434 | 8,963 | 18,526 |  |  |
| 11,404 | 29,702 | 9,942 | 23,022 | 17,290 | 15,571 |  |  |
| 11,903 | 20,284 | 9,579 | 16,453 | 15,462 | 28,061 |  |  |
| 8,855 | 15,970 | 9,807 | 27,994 | 12,286 | 20,567 |  |  |
| 8,391 | 20,036 | 12,425 | 21,307 | 8,978 | 24,020 |  |  |
| 12,391 | 22,797 | 10,881 | 31,194 | 9,001 | 21,825 |  |  |
| 9,966 | 18,559 | 18,403 | 57,410 | 8,388 | 24,280 |  |  |
| 11,956 | 22,562 | 9,135 | 20,037 | 9,344 | 29,804 |  |  |
| 9,322 | 29,194 | 14,898 | 22,547 | 8,338 | 15,246 |  |  |
| 9,384 | 21,070 | 14,987 | 17,284 | 8,491 | 14,816 |  |  |
| 8,848 | 24,209 | 14,403 | 40,489 | 10,147 | 28,251 |  |  |
| 9,457 | 18,540 | 18,745 | 23,070 | 8,351 | 40,131 |  |  |
| 11,351 | 26,357 | 13,074 | 17,092 | 9,653 | 33,626 |  |  |
| 8,784 | 32,049 | 12,877 | 17,007 | 9,760 | 24,641 |  |  |
| 10,928 | 20,299 | 9,507 | 14,919 | 12,001 | 19,605 |  |  |
| 8,422 | 32,376 | 12,390 | 17,194 | 9,845 | 29,280 |  |  |
| 8,351 | 26,846 | 12,147 | 17,719 | 10,218 | 37,368 |  |  |
| 12,362 | 19,734 | 9,812 | 14,851 | 9,193 | 23,483 |  |  |
| 8,104 | 22,357 | 11,377 | 28,241 | 9,868 | 20,972 |  |  |
| 8,627 | 21,444 | 11,203 | 20,259 | 9,997 | 20,251 |  |  |
| 12,213 | 17,815 | 10,022 | 25,041 | 9,594 | 23,208 |  |  |
| 9,317 | 28,191 | 9,532 | 21,430 | 9,656 | 24,353 |  |  |
| 9,370 | 26,254 | 19,081 | 18,660 | 10,538 | 25,502 |  |  |
| 9,590 | 18,711 | 16,169 | 22,483 | 9,144 | 19,591 |  |  |
| 11,709 | 18,488 | 10,617 | 21,447 | 11,329 | 22,455 |  |  |
| 12,076 | 20,568 | 8,953 | 18,870 | 9,418 | 20,988 |  |  |
| 9,177 | 21,180 | 14,755 | 23,700 | 11,918 | 18,829 |  |  |
| 13,185 | 29,214 | 10,091 | 20,913 | 12,724 | 23,168 |  |  |
| 9,819 | 22,076 | 13,159 | 25,078 | 13,163 | 26,766 |  |  |
| 10,132 | 25,027 | 8,511 | 19,143 | 11,697 | 21,649 |  |  |
| 10,127 | 19,205 | 12,024 | 19,999 | 9,777 | 21,889 |  |  |
| 8,855 | 17,138 | 9,568 | 18,948 | 11,249 | 26,722 |  |  |
| 9,439 | 31,209 | 9,994 | 20,758 | 13,537 | 25,194 |  |  |
| 8,903 | 37,826 | 11,898 | 20,627 | 9,884 | 20,076 |  |  |
| 8,467 | 22,506 | 19,110 | 23,123 | 9,338 | 27,300 |  |  |
| 9,724 | 22,011 | 12,422 | 21,146 | 12,573 | 22,092 |  |  |
| 8,510 | 28,182 | 10,745 | 23,466 | 13,254 | 24,705 |  |  |
| 14,750 | 43,980 | 11,242 | 20,690 | 8,182 | 36,098 |  |  |
| 9,175 | 14,962 | 12,479 | 25,314 | 10,670 | 29,559 |  |  |
| 11,133 | 27,138 | 9,365 | 21,767 | 11,899 | 30,729 |  |  |

| **Figure 2C** | | | |
| --- | --- | --- | --- |
| **0 nM** | **10 nM** | **100 nM** | **1000nM** |
| 100, | 87, | 255, | 438, |
| 100, | 71, | 239, | 460, |
| 100, | 120, | 317, | 295, |
| 100, | 298, | 448, | 253, |

| **Figure 2F** | | | |
| --- | --- | --- | --- |
| **0 nM** | **10 nM** | **100 nM** | **1000 nM** |
| 100, | 215, | 401, | 321, |
| 100, | 190, | 350, | 567, |
| 100, | 174, | 246, | 369, |
| 100, | 193, | 333, | 419, |

| **Figure 2G** | | | |
| --- | --- | --- | --- |
| **0 nM** | **10 nM** | **100 nM** | **1000 nM** |
| 100, | 127, | 213, | 199, |
| 100, | 93, | 187, | 298, |
| 100, | 91, | 181, | 240, |
| 100, | 110, | 194, | 246, |

| **Figure 3C** | | **Figure 3F** | |
| --- | --- | --- | --- |
| **0 STX/0 nM DHEAS** | **0 STX/100 nM DHEAS** | **10 nM STX, 0 nM DHEAS** | **10 nM STX, 100 nM DHEAS** |
| 14,930 | 15,338 | 18,305 | 22,253 |
| 13,902 | 15,072 | 12,566 | 18,839 |
| 11,594 | 12,088 | 11,220 | 18,494 |
| 12,335 | 14,091 | 10,629 | 20,302 |
| 14,112 | 21,432 | 12,911 | 17,858 |
| 10,429 | 17,978 | 15,805 | 22,007 |
| 10,684 | 16,266 | 14,363 | 19,514 |
| 9,967 | 29,525 | 15,324 | 23,256 |
| 10,174 | 26,315 | 17,757 | 23,634 |
| 9,285 | 23,084 | 12,490 | 19,780 |
| 10,554 | 23,718 | 10,848 | 25,146 |
| 10,502 | 23,513 | 11,767 | 40,679 |
| 8,398 | 31,996 | 13,289 | 20,633 |
| 9,010 | 27,667 | 11,765 | 28,244 |
| 10,387 | 33,400 | 12,800 | 39,249 |
| 11,502 | 23,287 | 11,073 | 29,662 |
| 10,736 | 26,862 | 10,312 | 26,065 |
| 8,393 | 26,295 | 9,446 | 17,414 |
| 9,420 | 21,246 | 8,855 | 16,093 |
| 8,649 | 21,340 | 9,594 | 21,482 |
| 8,874 | 17,209 | 8,645 | 28,805 |
| 8,269 | 27,715 | 11,309 | 39,994 |
| 9,364 | 26,533 | 10,822 | 29,740 |
| 9,136 | 23,089 | 14,390 | 33,817 |
| 9,737 | 19,296 | 11,409 | 21,232 |
| 9,905 | 28,160 | 12,782 | 24,115 |
| 10,241 | 25,464 | 12,742 | 27,279 |
| 10,861 | 22,612 | 11,762 | 25,334 |
| 11,186 | 18,207 | 11,420 | 31,855 |
| 8,910 | 34,093 | 13,005 | 23,077 |
| 8,995 | 21,962 | 11,319 | 30,503 |
| 8,157 | 32,062 | 13,424 | 33,851 |
| 10,399 | 15,144 | 11,231 | 21,378 |
| 9,143 | 16,282 | 10,244 | 27,246 |
| 9,085 | 29,124 | 9,852 | 28,648 |
| 9,837 | 25,000 | 8,571 | 17,425 |
| 10,226 | 24,188 | 11,768 | 23,325 |
| 8,355 | 29,763 | 13,071 | 23,614 |
| 10,432 | 28,169 | 12,220 | 21,439 |
| 10,047 | 27,037 | 8,944 | 24,455 |
| 9,379 | 28,068 | 15,487 | 30,587 |
| 9,206 | 24,663 | 9,683 | 28,431 |
| 9,019 | 17,541 | 13,799 | 18,160 |
| 10,600 | 23,690 | 11,290 | 23,185 |
| 12,021 | 17,933 | 10,659 | 16,715 |
| 11,582 | 23,271 | 11,991 | 22,929 |
| 11,006 | 19,522 | 9,720 | 18,517 |
| 9,872 | 30,911 | 12,387 | 21,496 |
| 10,241 | 18,509 | 9,678 | 18,823 |
| 12,570 | 20,123 | 11,539 | 23,691 |
| 10,583 | 26,945 | 13,761 | 31,585 |
| 8,956 | 16,109 | 12,952 | 21,429 |
| 10,918 | 17,087 | 12,148 | 18,720 |
| 10,438 | 15,869 | 12,087 | 16,555 |
| 11,317 | 22,065 | 11,052 | 19,857 |
| 10,716 | 22,418 | 10,698 | 17,052 |
| 12,781 | 19,323 | 9,682 | 18,293 |
| 12,163 | 26,632 | 9,944 | 27,081 |
| 11,317 | 29,460 | 9,603 | 36,454 |
| 13,424 | 27,388 | 10,680 | 26,310 |
| 11,457 | 23,862 | 10,632 | 28,370 |
| 10,128 | 27,188 | 9,362 | 31,959 |
| 10,649 | 22,903 | 13,897 | 17,649 |
| 12,815 | 23,099 | 11,715 | 26,298 |
| 13,270 | 23,941 | 11,696 | 23,033 |
| 10,105 | 20,939 | 9,274 | 33,925 |
| 11,992 | 20,927 | 10,489 | 28,267 |
| 10,536 | 26,956 | 10,648 | 24,059 |
| 11,471 | 24,937 | 13,495 | 22,783 |
| 9,814 | 25,324 | 13,225 | 35,057 |
| 9,620 | 18,892 | 10,958 | 34,145 |
| 11,584 | 23,107 | 10,733 | 48,286 |
| 14,218 | 18,720 | 11,155 | 32,300 |
| 10,998 | 27,319 | 13,403 | 24,936 |
| 9,146 | 29,032 | 13,394 | 25,313 |
| 9,106 | 24,336 | 9,851 | 31,030 |
| 11,326 | 22,785 | 9,434 | 37,037 |
| 10,497 | 17,657 | 9,895 | 28,591 |
| 14,002 | 23,230 | 13,179 | 28,424 |
| 10,716 | 22,116 | 11,360 | 29,922 |
| 10,524 | 18,648 | 9,601 | 28,445 |
| 11,393 | 17,152 | 13,392 | 27,050 |
| 10,635 | 23,606 | 11,728 | 33,520 |
| 12,755 | 31,803 | 13,133 | 19,329 |
| 13,112 | 21,410 | 12,762 | 26,049 |
| 11,033 | 19,850 | 13,654 | 25,267 |
| 10,215 | 19,579 | 13,463 | 35,532 |
| 10,278 | 21,417 | 10,730 | 28,125 |
| 9,325 | 22,720 | 10,809 | 23,102 |
| 13,891 | 26,368 | 11,673 | 17,074 |

| **Figure 4C** | | | |
| --- | --- | --- | --- |
| **0 nM** | **10 nM** | **100 nM** | **1000 nM** |
| 100, | 112, | 108, | 100, |
| 100, | 116, | 125, | 105, |
| 100, | 86, | 77, | 74, |
|  |  |  |  |

| **Figure 5E** | | | |
| --- | --- | --- | --- |
| **0 µM** | **1 µM** | **0 µM** | **1 µM** |
| 100, | 175, | 100, | 162, |
| 100, | 223, | 100, | 210, |
| 100, | 280, | 100, | 210, |
|  |  |  |  |

| **Figure 6C** | | **Figure 6F** | |
| --- | --- | --- | --- |
| **0 µM** | **1 µM** | **0 µM** | **1 µM** |
| 18,560 | 35,005 | 27,804 | 57,934 |
| 16,889 | 28,798 | 23,086 | 47,790 |
| 16,293 | 37,522 | 18,232 | 49,080 |
| 21,446 | 41,792 | 27,883 | 45,188 |
| 23,616 | 35,683 | 23,205 | 47,007 |
| 29,408 | 29,537 | 32,439 | 56,868 |
| 22,990 | 43,375 | 23,860 | 32,767 |
| 18,319 | 38,359 | 24,481 | 40,194 |
| 19,769 | 33,246 | 28,060 | 37,744 |
| 20,335 | 41,093 | 32,157 | 34,526 |
| 23,316 | 34,502 | 22,757 | 46,432 |
| 23,755 | 34,216 | 24,982 | 35,065 |
| 19,879 | 38,093 | 34,554 | 34,190 |
| 25,899 | 34,001 | 34,805 | 33,412 |
| 20,998 | 41,772 | 20,388 | 39,379 |
| 24,196 | 36,238 | 14,159 | 39,465 |
| 13,368 | 39,458 | 20,905 | 57,136 |
| 25,886 | 51,534 | 22,091 | 52,457 |
| 20,190 | 34,247 | 28,928 | 49,124 |
| 16,463 | 33,613 | 23,359 | 58,564 |
| 16,560 | 38,723 | 25,609 | 41,588 |
| 22,076 | 36,672 | 17,785 | 36,067 |
| 21,676 | 35,443 | 21,672 | 53,879 |
| 27,097 | 46,805 | 19,625 | 49,953 |
| 31,233 | 30,288 | 18,282 | 38,333 |
| 15,840 | 34,410 | 15,682 | 45,863 |
| 22,660 | 36,038 | 21,349 | 45,086 |
| 9,167 | 39,787 | 20,292 | 55,388 |
| 18,336 | 25,732 | 26,021 | 54,193 |
| 18,992 | 44,952 | 23,738 | 50,051 |
| 21,358 | 48,706 | 27,126 | 38,948 |
| 20,687 | 36,990 | 18,466 | 50,228 |
| 18,807 | 34,814 | 29,021 | 37,991 |
| 18,255 | 32,751 | 10,235 | 32,990 |
| 19,089 | 25,546 | 18,814 | 38,203 |
| 17,556 | 30,684 | 21,209 | 49,716 |
| 16,089 | 38,257 | 21,431 | 41,139 |
| 14,010 | 32,826 | 17,560 | 39,973 |
| 13,732 | 33,911 | 24,902 | 32,005 |
| 10,371 | 49,898 | 22,015 | 39,187 |
| 18,559 | 42,674 | 28,027 | 26,727 |
| 12,443 | 41,917 | 23,745 | 27,016 |
| 19,207 | 31,612 | 23,621 | 28,892 |
| 17,150 | 40,822 | 16,125 | 22,533 |
| 13,386 | 40,781 | 20,482 | 30,124 |

| **Figure 7C** | | **Figure 7F** | | **Figure 7I** | | **Figure 7L** | |
| --- | --- | --- | --- | --- | --- | --- | --- |
| **0 µM** | **1 µM** | **0 µM** | **1 µM** | **0 µM** | **1 µM** | **0 µM** | **1 µM** |
| 18,27 | 37,81 | 21,01 | 46,79 | 29,46 | 35,54 | 35,00 | 59,35 |
| 28,94 | 40,23 | 19,23 | 33,46 | 31,75 | 40,06 | 28,35 | 43,13 |
| 24,06 | 43,62 | 15,75 | 44,95 | 25,45 | 42,08 | 21,03 | 43,58 |
| 16,36 | 48,51 | 19,18 | 43,68 | 30,00 | 40,47 | 14,33 | 45,16 |
| 21,65 | 57,03 | 16,75 | 36,25 | 20,29 | 36,67 | 24,07 | 35,25 |
| 16,86 | 41,93 | 19,75 | 41,20 | 16,99 | 36,15 | 25,87 | 36,93 |
| 17,16 | 38,38 | 15,02 | 37,86 | 20,95 | 44,09 | 21,65 | 37,95 |
| 20,81 | 38,25 | 16,54 | 44,06 | 19,36 | 37,78 | 25,29 | 34,70 |
| 22,11 | 58,36 | 18,93 | 38,01 | 31,29 | 31,48 | 25,34 | 31,16 |
| 14,36 | 46,93 | 18,92 | 27,00 | 22,99 | 34,55 | 33,39 | 28,26 |
| 21,43 | 49,44 | 23,55 | 30,61 | 27,99 | 48,92 | 26,57 | 34,81 |
| 15,24 | 48,54 | 17,32 | 45,80 | 20,53 | 46,54 | 19,87 | 28,36 |
| 26,05 | 33,91 | 20,25 | 42,01 | 18,73 | 31,74 | 25,75 | 34,79 |
| 18,59 | 53,27 | 20,04 | 38,28 | 19,34 | 41,95 | 17,65 | 34,14 |
| 20,64 | 35,35 | 20,89 | 28,50 | 19,04 | 39,31 | 24,77 | 47,31 |
| 17,96 | 38,18 | 22,54 | 34,18 | 17,55 | 45,11 | 20,30 | 59,55 |
| 17,95 | 38,23 | 12,80 | 34,42 | 20,06 | 50,26 | 27,04 | 43,69 |
| 21,15 | 54,65 | 18,37 | 37,16 | 19,58 | 43,67 | 24,13 | 55,08 |
| 14,25 | 45,77 | 18,45 | 32,17 | 21,41 | 52,85 | 20,22 | 62,64 |
| 10,95 | 38,74 | 19,45 | 27,98 | 20,23 | 54,44 | 25,78 | 50,96 |
| 13,26 | 33,52 | 18,70 | 45,90 | 21,90 | 53,40 | 28,02 | 45,10 |
| 13,85 | 35,08 | 21,72 | 59,04 | 20,59 | 40,15 | 22,52 | 44,01 |
| 13,96 | 16,11 | 19,83 | 31,03 | 22,52 | 45,88 | 25,24 | 29,23 |
| 9,59 | 36,64 | 28,28 | 34,92 | 23,67 | 35,80 | 21,91 | 34,26 |
| 14,57 | 44,58 | 26,66 | 34,92 | 24,76 | 41,14 | 27,80 | 26,53 |
| 15,46 | 47,53 | 19,51 | 39,91 | 16,01 | 45,75 | 31,13 | 31,74 |
| 18,18 | 47,59 | 21,43 | 54,00 | 19,86 | 58,11 | 29,45 | 41,78 |
| 16,30 | 51,33 | 18,21 | 33,00 | 16,95 | 47,23 | 30,60 | 21,92 |
| 21,27 | 22,28 | 16,98 | 33,03 | 19,31 | 38,93 | 24,76 | 40,28 |
| 16,38 | 36,79 | 16,48 | 25,81 | 18,33 | 40,72 | 24,79 | 37,60 |
| 14,06 | 37,17 | 16,47 | 26,54 | 14,95 | 43,04 | 19,42 | 23,21 |
| 16,18 | 37,87 | 16,57 | 31,94 | 11,90 | 49,38 | 14,42 | 28,94 |
| 28,60 | 22,22 | 18,97 | 41,49 | 17,10 | 47,17 | 27,23 | 47,74 |
| 22,44 | 26,01 | 22,50 | 42,70 | 18,78 | 37,13 | 19,20 | 43,75 |
| 24,44 | 34,14 | 24,42 | 40,63 | 13,48 | 33,20 | 18,53 | 31,73 |
| 15,17 | 35,18 | 23,56 | 26,68 | 17,89 | 32,29 | 22,24 | 20,27 |
| 19,64 | 24,68 | 18,31 | 25,14 | 17,61 | 18,16 | 17,77 | 36,06 |
| 17,92 | 36,19 | 12,29 | 29,23 | 20,26 | 30,51 | 14,36 | 47,19 |
| 13,85 | 35,23 | 21,13 | 33,64 | 17,71 | 47,66 | 20,31 | 53,24 |
| 20,56 | 38,72 | 33,44 | 35,54 | 11,70 | 23,72 | 13,25 | 37,24 |
| 17,16 | 47,86 | 27,17 | 45,88 | 15,73 | 27,11 | 18,04 | 25,32 |
| 18,34 | 34,49 | 24,27 | 39,22 | 19,92 | 46,34 | 16,23 | 43,05 |
| 18,86 | 35,28 | 24,30 | 38,07 | 13,33 | 39,23 | 15,18 | 45,96 |
| 25,29 | 30,58 | 23,60 | 34,35 | 18,76 | 45,54 | 16,69 | 52,99 |
| 18,54 | 58,84 | 19,00 | 45,68 | 23,49 | 36,09 | 11,94 | 33,55 |

| **Figure 8A** | | | | | | | | |
| --- | --- | --- | --- | --- | --- | --- | --- | --- |
| **Day** | **0 µM DHEAS** | | | | **1 µM DHEAS** | | | |
| 0, | 13,8 | 10,2 | 16,8 | 6,6 | 6,0 | 15,6 | 10,8 | 10,2 |
| 1, | 11,4 | 12,6 | 16,8 | 18,6 | 24,2 | 19,8 | 22,8 | 20,4 |
| 2, | 23,4 | 17,4 | 22,8 | 22,2 | 35,2 | 33,0 | 30,0 | 28,2 |

| **Figure 10B** | | | | | |
| --- | --- | --- | --- | --- | --- |
| **0 µM** | **1 µM** | **0 µM** | **1 µM** | **0 µM** | **1 µM** |
| 8,000 | 29,912 | 9,605 | 25,317 | 9,341 | 22,603 |
| 8,026 | 42,234 | 8,742 | 18,049 | 8,516 | 15,616 |
| 8,107 | 41,980 | 8,430 | 14,967 | 8,077 | 13,570 |
| 8,000 | 37,216 | 8,990 | 15,510 | 9,821 | 20,589 |
| 8,031 | 29,465 | 8,501 | 24,021 | 12,108 | 31,906 |
| 8,155 | 39,351 | 8,971 | 19,878 | 11,366 | 24,133 |
| 12,260 | 26,330 | 12,604 | 22,624 | 8,802 | 25,745 |
| 10,238 | 16,889 | 9,813 | 20,617 | 9,144 | 14,814 |
| 11,395 | 24,610 | 8,646 | 16,325 | 10,051 | 25,825 |
| 10,833 | 25,246 | 8,247 | 15,208 | 12,651 | 28,442 |
| 8,717 | 27,660 | 10,613 | 24,469 | 13,128 | 19,730 |
| 9,015 | 27,586 | 10,605 | 14,620 | 7,987 | 21,366 |
| 8,862 | 21,092 | 13,827 | 14,950 | 9,250 | 31,510 |
| 8,880 | 13,499 | 13,012 | 25,179 | 9,674 | 25,381 |
| 12,467 | 14,241 | 10,761 | 21,256 | 10,588 | 12,742 |
| 11,925 | 18,847 | 10,032 | 16,536 | 9,685 | 13,498 |
| 8,663 | 22,322 | 10,991 | 23,509 | 8,253 | 23,992 |
| 8,793 | 21,996 | 8,204 | 17,774 | 8,371 | 27,194 |
| 9,792 | 17,506 | 8,448 | 22,357 | 9,629 | 17,720 |
| 8,635 | 28,591 | 8,872 | 24,716 | 10,689 | 16,568 |
| 8,341 | 20,706 | 10,138 | 29,650 | 9,776 | 24,679 |
| 9,736 | 28,908 | 17,489 | 23,539 | 8,387 | 16,649 |
| 8,821 | 23,228 | 10,119 | 22,979 | 8,324 | 19,175 |
| 8,678 | 31,589 | 22,978 | 26,011 | 11,782 | 18,781 |
| 8,246 | 33,472 | 10,078 | 27,974 | 11,179 | 15,487 |
| 8,179 | 24,072 | 9,017 | 22,495 | 8,966 | 22,514 |
| 8,031 | 27,531 | 9,342 | 15,416 | 9,001 | 26,419 |
| 8,227 | 23,402 | 8,207 | 28,347 | 9,546 | 23,385 |
| 8,591 | 17,737 | 8,150 | 22,571 | 10,726 | 16,518 |
| 9,001 | 18,145 | 8,510 | 19,624 | 8,553 | 23,675 |
| 8,308 | 13,074 | 11,734 | 24,058 | 13,789 | 16,332 |
| 8,425 | 19,851 | 9,780 | 13,244 | 12,042 | 19,693 |
| 8,302 | 20,764 | 8,777 | 20,232 | 9,794 | 11,039 |
| 8,180 | 22,136 | 8,714 | 21,058 | 9,053 | 10,974 |
| 8,523 | 18,212 | 11,436 | 33,213 | 10,019 | 19,547 |
| 10,656 | 21,495 | 9,899 | 22,482 | 9,405 | 9,689 |
| 9,427 | 23,611 | 10,980 | 21,084 | 39,671 | 19,917 |
| 10,059 | 17,675 | 10,623 | 11,727 | 10,045 | 24,248 |
| 8,168 | 23,121 | 10,468 | 15,427 | 8,547 | 26,104 |
| 8,415 | 31,882 | 8,284 | 15,884 | 9,882 | 16,699 |
| 8,000 | 43,654 | 8,978 | 14,708 | 9,881 | 16,818 |
| 9,367 | 26,138 | 9,423 | 29,915 | 10,909 | 12,167 |
| 10,253 | 27,275 | 8,479 | 21,459 | 11,379 | 22,326 |
| 10,799 | 25,799 | 8,226 | 18,191 | 11,168 | 26,925 |
| 8,334 | 22,739 | 8,408 | 16,774 | 10,294 | 32,557 |
| 8,621 | 24,039 | 8,874 | 18,056 | 10,775 | 31,340 |
| 8,286 | 21,276 | 10,275 | 15,161 | 12,049 | 24,307 |
| 8,431 | 22,999 | 8,746 | 9,860 | 10,998 | 14,554 |
| 8,385 | 29,084 | 9,572 | 17,870 | 11,007 | 21,511 |
| 8,524 | 32,878 | 8,660 | 15,347 | 9,345 | 14,449 |
| 9,251 | 20,721 | 8,920 | 27,532 | 9,052 | 23,281 |
| 8,951 | 16,095 | 9,515 | 21,623 | 10,746 | 26,322 |
| 10,485 | 19,218 | 9,134 | 24,764 | 10,407 | 27,449 |
| 8,411 | 17,159 | 8,470 | 18,088 | 10,372 | 23,875 |
| 9,474 | 23,555 | 12,099 | 30,809 | 8,537 | 20,319 |
| 8,631 | 18,239 | 8,110 | 20,851 | 8,680 | 21,172 |
| 9,659 | 23,057 | 8,954 | 24,959 | 8,563 | 10,519 |
| 8,205 | 39,313 | 10,292 | 13,562 | 8,949 | 10,570 |
| 8,940 | 33,842 | 7,948 | 22,276 | 9,524 | 22,228 |
| 8,877 | 27,958 | 9,309 | 21,132 | 9,868 | 12,909 |
| 9,852 | 29,298 | 7,946 | 26,664 | 9,937 | 10,463 |
| 8,698 | 26,794 | 8,246 | 17,772 | 10,335 | 19,330 |
| 11,227 | 19,452 | 10,368 | 21,731 | 10,699 | 11,573 |
| 10,559 | 25,411 | 8,219 | 20,955 | 9,867 | 29,862 |
| 10,838 | 18,775 | 8,473 | 14,718 | 8,307 | 34,782 |
| 8,882 | 24,512 | 7,926 | 13,963 | 8,371 | 9,704 |
| 9,376 | 27,580 | 9,132 | 17,698 | 9,673 | 17,337 |
| 8,805 | 36,847 | 8,150 | 18,338 | 9,748 | 15,701 |
| 10,925 | 28,109 | 9,844 | 18,777 | 9,685 | 17,449 |
| 9,461 | 36,246 | 9,385 | 12,516 | 10,686 | 13,529 |
| 8,664 | 36,962 | 8,344 | 17,585 | 11,901 | 22,237 |
| 10,834 | 29,424 | 8,205 | 15,390 | 9,244 | 15,454 |
| 8,796 | 25,648 | 8,165 | 13,933 | 13,132 | 10,932 |
| 10,493 | 26,480 | 8,254 | 10,616 | 10,042 | 12,362 |
| 8,702 | 30,429 | 8,470 | 11,569 | 12,658 | 19,709 |
| 10,020 | 37,932 | 8,642 | 15,705 | 12,158 | 23,220 |
| 11,550 | 37,184 | 10,332 | 12,078 | 12,909 | 12,362 |
| 10,536 | 34,862 | 11,025 | 22,767 | 9,174 | 15,832 |
| 10,244 | 33,045 | 9,445 | 16,847 | 9,230 | 15,260 |
| 8,266 | 29,904 | 8,455 | 22,473 | 10,051 | 32,075 |
| 8,298 | 30,030 | 9,121 | 19,958 | 13,060 | 13,344 |
| 8,189 | 29,310 | 8,963 | 18,337 | 16,035 | 21,165 |
| 8,011 | 29,339 | 8,189 | 24,866 | 9,202 | 17,064 |
| 8,189 | 45,487 | 8,563 | 36,894 | 10,304 | 15,152 |
| 9,069 | 21,659 | 8,706 | 33,146 | 11,172 | 17,781 |
| 8,415 | 37,053 | 9,456 | 22,768 | 11,447 | 28,259 |
| 8,223 | 40,566 | 9,674 | 18,066 | 13,422 | 21,674 |
| 8,301 | 35,389 | 8,507 | 30,883 | 11,126 | 26,033 |
| 8,427 | 31,022 | 8,313 | 24,839 | 11,239 | 20,981 |
| 8,190 | 38,455 | 11,019 | 13,929 | 9,818 | 19,979 |
| 8,000 | 29,912 | 9,605 | 25,317 | 9,341 | 22,603 |
| 8,026 | 42,234 | 8,742 | 18,049 | 8,516 | 15,616 |
| 8,107 | 41,980 | 8,430 | 14,967 | 8,077 | 13,570 |
| 8,000 | 37,216 | 8,990 | 15,510 | 9,821 | 20,589 |
| 8,031 | 29,465 | 8,501 | 24,021 | 12,108 | 31,906 |

| **Figure 10D** | | | | | |
| --- | --- | --- | --- | --- | --- |
| **0 µM** | **1 µM** | **0 µM** | **1 µM** | **0 µM** | **1 µM** |
| 11,219 | 11,907 | 11,677 | 9,046 | 12,130 | 11,954 |
| 11,219 | 10,022 | 12,428 | 11,065 | 9,566 | 10,694 |
| 9,190 | 8,617 | 10,400 | 9,848 | 12,783 | 11,678 |
| 9,394 | 9,359 | 8,720 | 21,965 | 13,656 | 8,849 |
| 9,320 | 10,156 | 9,261 | 9,778 | 17,591 | 11,250 |
| 9,904 | 9,310 | 9,175 | 8,902 | 10,111 | 8,567 |
| 9,934 | 9,560 | 9,464 | 9,548 | 10,325 | 11,176 |
| 10,277 | 9,238 | 9,528 | 9,083 | 8,654 | 12,899 |
| 9,609 | 9,184 | 8,327 | 8,615 | 15,704 | 9,932 |
| 8,571 | 9,816 | 8,344 | 11,004 | 9,605 | 9,656 |
| 8,663 | 11,029 | 8,583 | 9,506 | 11,768 | 9,067 |
| 12,667 | 11,561 | 9,416 | 9,974 | 8,516 | 12,191 |
| 8,634 | 9,219 | 19,533 | 8,434 | 8,406 | 11,789 |
| 11,193 | 8,841 | 10,030 | 12,166 | 9,885 | 9,141 |
| 9,171 | 8,787 | 8,682 | 16,674 | 8,947 | 8,787 |
| 8,094 | 8,486 | 9,630 | 10,468 | 9,583 | 9,389 |
| 8,585 | 8,474 | 11,013 | 8,986 | 8,415 | 10,208 |
| 8,286 | 8,451 | 10,479 | 10,516 | 8,960 | 8,988 |
| 11,346 | 9,860 | 8,925 | 10,016 | 9,656 | 8,679 |
| 9,469 | 10,280 | 8,380 | 9,207 | 8,282 | 15,737 |
| 9,777 | 9,977 | 8,552 | 10,103 | 9,611 | 11,577 |
| 9,789 | 10,011 | 8,072 | 9,829 | 11,003 | 9,979 |
| 10,486 | 9,382 | 9,219 | 10,839 | 37,219 | 8,611 |
| 8,486 | 10,224 | 10,267 | 9,112 | 8,814 | 9,535 |
| 8,464 | 8,474 | 12,472 | 8,965 | 10,435 | 11,008 |
| 9,118 | 9,206 | 8,867 | 9,720 | 8,291 | 10,473 |
| 8,556 | 8,696 | 9,096 | 9,428 | 8,469 | 12,289 |
| 9,178 | 9,679 | 8,341 | 8,766 | 9,520 | 15,035 |
| 10,720 | 9,791 | 10,288 | 8,896 | 9,050 | 10,016 |
| 9,033 | 9,089 | 9,334 | 10,338 | 9,561 | 9,774 |
| 8,383 | 9,531 | 9,127 | 9,006 | 8,399 | 9,112 |
| 9,506 | 8,831 | 9,159 | 10,778 | 10,254 | 9,652 |
| 8,169 | 9,104 | 9,603 | 9,801 | 9,664 | 9,251 |
| 8,540 | 9,540 | 10,127 | 9,180 | 10,190 | 11,426 |
| 8,462 | 11,434 | 9,632 | 9,119 | 8,373 | 14,350 |
| 9,162 | 12,287 | 8,713 | 9,083 | 9,052 | 11,584 |
| 8,297 | 11,581 | 9,071 | 9,114 | 9,919 | 10,513 |
| 8,570 | 10,482 | 9,588 | 12,846 | 11,573 | 8,921 |
| 8,695 | 12,415 | 9,835 | 11,351 | 11,661 | 14,972 |
| 8,055 | 8,525 | 11,179 | 8,453 | 9,088 | 13,272 |
| 8,685 | 10,643 | 11,332 | 8,961 | 8,687 | 13,261 |
| 8,789 | 9,972 | 10,136 | 12,269 | 9,300 | 12,896 |
| 8,746 | 10,758 | 10,545 | 9,356 | 8,811 | 15,236 |
| 8,809 | 10,064 | 10,002 | 9,130 | 11,458 | 9,125 |
| 8,512 | 8,841 | 9,075 | 11,143 | 12,139 | 19,750 |
| 8,369 | 8,761 | 10,263 | 12,636 | 10,099 | 10,755 |
| 8,433 | 10,692 | 9,440 | 11,201 | 12,259 | 17,386 |
| 8,479 | 9,673 | 9,012 | 10,031 | 10,142 | 16,117 |
| 8,617 | 10,184 | 9,314 | 10,333 | 10,285 | 10,211 |
| 10,211 | 8,901 | 8,486 | 9,989 | 10,199 | 18,268 |
| 11,036 | 9,601 | 8,615 | 12,270 | 8,782 | 14,344 |
| 9,185 | 8,387 | 9,514 | 13,902 | 8,982 | 10,637 |
| 8,371 | 8,319 | 9,602 | 9,774 | 7,988 | 10,198 |
| 10,509 | 8,973 | 11,322 | 11,269 | 8,196 | 10,467 |
| 8,418 | 10,078 | 22,387 | 8,591 | 9,174 | 8,794 |
| 9,437 | 10,604 | 11,041 | 11,447 | 11,704 | 11,140 |
| 8,639 | 10,823 | 10,886 | 10,102 | 8,490 | 10,353 |
| 8,323 | 8,804 | 8,460 | 15,550 | 11,473 | 9,905 |
| 9,874 | 9,064 | 9,075 | 9,842 | 11,914 | 9,163 |
| 9,769 | 9,704 | 8,695 | 10,328 | 9,997 | 9,374 |
| 9,635 | 8,818 | 10,255 | 10,885 | 8,953 | 15,201 |
| 9,847 | 8,948 | 8,042 | 9,435 | 10,462 | 10,209 |
| 8,897 | 10,043 | 8,165 | 9,208 | 9,517 | 11,791 |
| 8,924 | 9,174 | 8,691 | 9,676 | 10,775 | 12,469 |
| 11,155 | 9,230 | 9,104 | 9,974 | 10,186 | 9,103 |
| 9,543 | 8,228 | 9,025 | 10,219 | 9,903 | 9,621 |
| 11,467 | 10,210 | 17,212 | 10,043 | 8,844 | 9,951 |
| 9,866 | 9,483 | 8,593 | 8,519 | 10,328 | 8,787 |
| 13,030 | 10,105 | 8,740 | 8,806 | 11,456 | 9,345 |
| 10,368 | 10,262 | 11,914 | 14,343 | 9,980 | 14,408 |
| 10,208 | 11,566 | 8,689 | 11,722 | 9,626 | 11,326 |
| 10,618 | 10,329 | 9,370 | 9,934 | 10,859 | 11,736 |
| 8,843 | 11,993 | 10,210 | 9,813 | 9,374 | 11,059 |
| 8,569 | 11,725 | 8,943 | 12,454 | 9,454 | 8,901 |
| 10,207 | 11,774 | 8,575 | 9,027 | 9,028 | 14,994 |
| 8,078 | 10,663 | 8,679 | 9,607 | 8,448 | 9,425 |
| 8,573 | 11,241 | 9,792 | 8,997 | 11,313 | 9,302 |
| 8,853 | 8,798 | 8,830 | 10,722 | 8,405 | 9,651 |
| 9,238 | 9,357 | 10,266 | 10,049 | 12,611 | 10,296 |
| 8,797 | 9,582 | 8,808 | 10,396 | 10,155 | 15,526 |
| 8,796 | 9,623 | 8,719 | 10,955 | 11,451 | 9,696 |
| 9,978 | 10,183 | 8,712 | 8,651 | 9,112 | 16,722 |
| 8,319 | 10,078 | 8,319 | 9,398 | 9,706 | 14,258 |
| 9,368 | 9,203 | 8,955 | 11,995 | 13,513 | 14,784 |
| 9,115 | 8,539 | 9,323 | 11,148 | 12,285 | 16,207 |
| 8,941 | 10,135 | 9,673 | 9,138 | 10,591 | 14,340 |
| 9,184 | 9,328 | 10,150 | 9,632 | 9,052 | 18,389 |
| 11,564 | 10,137 | 8,099 | 9,337 | 8,870 | 14,979 |
| 10,805 | 8,888 | 8,072 | 11,901 | 8,877 | 13,187 |
| 11,305 | 10,267 | 9,511 | 8,764 | 8,386 | 12,921 |

| **Figure 11C** | | | |
| --- | --- | --- | --- |
| **0 µM** | **1 µM** | **0 µM** | **1 µM** |
| 100, | 324, | 100, | 89, |
| 100, | 289, | 100, | 92, |
| 100, | 312, | 100, | 120, |
|  |  |  |  |

| **Figure 12C** | |  | |  | |  | |
| --- | --- | --- | --- | --- | --- | --- | --- |
| **0 µM** | **1 µM** | **0 µM** | **1 µM** | **0 µM** | **1 µM** | **0 µM** | **1 µM** |
| 18,157 | 36,771 | 20,353 | 43,244 | 23,402 | 22,794 | 20,491 | 24,556 |
| 13,785 | 34,066 | 22,085 | 45,344 | 18,898 | 23,680 | 22,815 | 30,113 |
| 14,324 | 32,358 | 23,580 | 40,249 | 17,235 | 24,698 | 22,548 | 26,562 |
| 15,733 | 32,092 | 27,754 | 39,176 | 19,082 | 24,585 | 14,414 | 23,333 |
| 11,069 | 25,962 | 25,881 | 38,358 | 26,046 | 25,693 | 20,112 | 28,392 |
| 16,787 | 27,281 | 23,554 | 33,213 | 22,956 | 17,665 | 21,238 | 30,674 |
| 10,574 | 35,831 | 29,224 | 33,913 | 22,344 | 21,418 | 24,189 | 30,133 |
| 12,856 | 43,828 | 17,041 | 40,007 | 20,559 | 24,866 | 17,131 | 25,489 |
| 14,321 | 46,177 | 24,826 | 34,153 | 16,319 | 28,257 | 16,318 | 25,683 |
| 21,634 | 34,863 | 21,680 | 40,813 | 20,048 | 24,274 | 19,995 | 26,331 |
| 21,953 | 23,742 | 22,386 | 37,407 | 23,266 | 20,276 | 20,683 | 25,693 |
| 13,496 | 33,529 | 21,674 | 41,048 | 16,483 | 18,156 | 18,443 | 32,653 |
| 23,758 | 39,652 | 20,739 | 39,767 | 17,631 | 25,750 | 21,593 | 43,959 |
| 19,094 | 44,359 | 21,871 | 51,215 | 21,635 | 25,088 | 26,164 | 46,474 |
| 16,794 | 37,047 | 16,707 | 55,257 | 18,961 | 27,242 | 17,215 | 26,234 |
| 11,865 | 35,415 | 19,740 | 38,742 | 17,960 | 19,326 | 17,570 | 20,376 |
| 14,371 | 39,866 | 16,403 | 50,079 | 20,462 | 22,774 | 16,230 | 25,759 |
| 11,549 | 38,919 | 23,121 | 45,799 | 25,298 | 18,813 | 17,857 | 28,155 |
| 11,760 | 38,020 | 18,477 | 41,079 | 24,640 | 18,644 | 24,935 | 29,556 |
| 14,538 | 40,514 | 14,510 | 49,415 | 15,660 | 12,702 | 18,363 | 26,228 |
| 19,935 | 33,052 | 18,073 | 49,260 | 23,132 | 25,654 | 18,379 | 23,101 |
| 11,696 | 33,965 | 15,936 | 39,941 | 17,423 | 24,226 | 26,563 | 17,194 |
| 15,934 | 48,806 | 17,030 | 48,524 | 20,588 | 26,318 | 27,490 | 18,310 |
| 14,018 | 35,829 | 17,540 | 37,216 | 19,035 | 22,485 | 23,298 | 15,539 |
| 14,617 | 44,304 | 20,191 | 39,733 | 16,500 | 21,252 | 24,783 | 15,946 |
| 13,909 | 45,778 | 18,674 | 40,290 | 15,623 | 25,244 | 21,568 | 17,534 |
| 12,367 | 42,055 | 17,337 | 37,143 | 20,406 | 21,230 | 20,329 | 20,197 |
| 14,751 | 35,283 | 17,979 | 56,283 | 20,534 | 20,730 | 23,526 | 22,897 |
| 14,876 | 30,976 | 17,845 | 34,030 | 21,597 | 25,897 | 23,384 | 20,071 |
| 14,847 | 38,815 | 12,909 | 32,486 | 17,979 | 23,359 | 26,705 | 21,523 |
| 16,999 | 44,664 | 18,762 | 35,095 | 20,608 | 26,393 | 17,050 | 12,955 |
| 14,187 | 37,726 | 20,958 | 28,811 | 20,083 | 26,443 | 18,140 | 25,901 |
| 14,553 | 37,877 | 17,896 | 40,075 | 18,960 | 26,449 | 18,003 | 14,508 |
| 23,249 | 34,967 | 18,555 | 37,139 | 19,436 | 29,464 | 17,669 | 19,225 |
| 58,978 | 44,691 | 19,838 | 35,163 | 17,838 | 24,744 | 15,961 | 32,705 |
| 25,984 | 56,362 | 19,905 | 29,244 | 21,555 | 27,703 | 14,461 | 23,084 |
| 53,358 | 43,690 | 19,137 | 36,208 | 16,537 | 29,821 | 18,166 | 16,691 |
| 20,189 | 32,574 | 24,230 | 33,253 | 16,116 | 30,312 | 22,099 | 16,331 |
| 24,080 | 42,588 | 18,249 | 25,216 | 18,583 | 28,501 | 20,847 | 16,849 |
| 13,773 | 39,175 | 20,055 | 35,888 | 21,206 | 26,537 | 23,526 | 16,848 |
| 14,996 | 40,408 | 22,700 | 36,593 | 24,528 | 23,755 | 25,074 | 17,429 |
| 16,238 | 39,727 | 19,492 | 48,394 | 22,697 | 30,801 | 23,330 | 26,144 |
| 19,180 | 39,821 | 20,598 | 36,781 | 17,483 | 22,703 | 23,670 | 22,655 |
| 22,999 | 47,907 | 19,484 | 55,705 | 15,099 | 16,702 | 22,467 | 27,828 |
| 22,133 | 47,146 | 18,341 | 46,118 | 15,320 | 17,773 | 15,286 | 34,121 |

| **Figure 13A** | | | | | | |
| --- | --- | --- | --- | --- | --- | --- |
| **Day** | **0 µM DHEAS** | | | **1 µM DHEAS** | | |
| **0** | 16,8 | 17,8 | 15,8 | 14,4 | 15,0 | 13,8 |
| **1** | 26,4 | 27,6 | 25,2 | 39,0 | 41,2 | 36,8 |
| **2** | 31,2 | 30,5 | 29,9 | 53,7 | 51,1 | 55,4 |

| **Figure 13C** | | | | | | | | |
| --- | --- | --- | --- | --- | --- | --- | --- | --- |
| **Day** | **0 µM DHEAS** | | | | **1 µM DHEAS** | | | |
| 0, | 18,0 | 18,0 | 17,4 | 20,4 | 18,0 | 20,4 | 16,2 | 13,2 |
| 1, | 19,2 | 28,2 | 28,2 | 25,6 | 23,4 | 25,6 | 33,0 | 29,4 |
| 2, | 25,8 | 33,6 | 31,8 | 27,6 | 29,4 | 31,8 | 38,4 | 33,0 |
